# Footprint preparation with nanofractures in a supraspinatus repair cuts in half the retear rate at 1-year follow-up. A randomized controlled trial

**DOI:** 10.1007/s00167-020-06073-7

**Published:** 2020-06-01

**Authors:** Miguel Angel Ruiz Ibán, Eduardo Sanchez Alepuz, Jorge Diaz Heredia, Abdul-ilah Hachem, Leon Ezagüi Bentolila, Angel Calvo, Carlos Verdú, Ignacio de Rus Aznar, Francesc Soler Romagosa

**Affiliations:** 1 Unidad de Hombro y Codo, Hospital Universitario Ramón y Cajal, Cta Colmenar km 9,100, 28046 Madrid, Spain; 2 Hospital IMED Valencia, Valencia, Spain; 3 Head of the Shoulder Unit, Hospital Universitario de Bellvitge, Barcelona, Spain; 4 Hospital Egarsat, Barcelona, Spain; 5 Arthrosport, Zaragoza, Spain; 6 Unidad de Hombro y Codo, Hospital General Universitario de Elche, Elche, Alicante Spain; 7 Traumadvance, Terrassa, Barcelona, Spain

**Keywords:** Shoulder arthroscopy, Rotator cuff, Rotator cuff repair, Microfracture, Footprint preparation, Supraspinatus tear, Shoulder, Rotator cuff retear

## Abstract

**Purpose:**

To evaluate if adding nanofractures to the footprint of a supraspinatus tear repair would have any effect in the outcomes at one-year follow-up.

**Methods:**

Multicentric, triple-blinded, randomized trial with 12-months follow-up. Subjects with isolated symptomatic reparable supraspinatus tears smaller than 3 cm and without grade 4 fatty infiltration were included. These were randomized to two groups: In the Control group an arthroscopic supraspinatus repair was performed; in the Nanofracture group the footprint was additionally prepared with nanofractures (1 mm wide, 9 mm deep microfractures). Clinical evaluation was done with Constant score, EQ-5D-3L, and Brief Pain Inventory. The primary outcome was the retear rate in MRI at 12-months follow-up. Secondary outcomes were: characteristics of the retear (at the footprint or at the musculotendinous junction) and clinical outcomes.

**Results:**

Seventy-one subjects were randomized. Two were lost to follow-up, leaving 69 participants available for assessment at 12-months follow-up (33 in the Control group and 36 in the Nanofracture Group). The Nanofracture group had lower retear rates than the Control group (7/36 [19.4%] vs 14/33 [42.4%], differences significant, *p* = 0.038). Retear rates at the musculotendinous junction were similar but the Nanofracture group had better tendon healing rates to the bone (34/36 [94.4%] vs. 24/33 [66.71%], *p* = 0.014). Clinically both groups had significant improvements, but no differences were found between groups.

**Conclusion:**

Adding nanofractures at the footprint during an isolated supraspinatus repair lowers in half the retear rate at 12-months follow-up. This is due to improved healing at the footprint.

**Level of evidence:**

Level I.

**Electronic supplementary material:**

The online version of this article (10.1007/s00167-020-06073-7) contains supplementary material, which is available to authorized users.

## Introduction

Rotator cuff tears are one of the most frequent causes of pain and disfunction in the older adult population [13]. Although many rotator cuff tears can be dealt conservatively, some of them require surgical repair [1]. Despite advances in surgical technique, the retear rates are often over 20% [10]. This high incidence of repair failure is due to fundamental biological issues related to the difficulty to obtain consistent tendon-to-bone healing and the unstoppable development of tendon degeneration [4]. Many efforts have been made to increase the chances to attain healing. Mechanical advances in repair techniques (using double row or transosseous equivalent [TOE] suture configurations) have got only limited benefits. Improving the biological environment using platelet-rich plasma [11] or stem cells [20] might increase the healing rate marginally.

It has been suggested that performing small holes (microfractures/bone marrow stimulation) in the bone footprint before repair might allow for an increased local outflow of stem cells and other factors that might improve the healing rate [5, 18]. Despite these, at least two randomized controlled trials [14, 15] have not found any significant effect on cuff healing. It has been suggested that performing deeper and thinner holes in the bone (nanofractures) would allow for an increased biological response [6] but this is unproven clinically.

The objective of this randomized controlled trial was to evaluate if adding nanofractures (1 mm wide, 9 mm deep, microfractures) at the footprint of a supraspinatus tear repair would have any effect in the radiological and clinical outcomes at one-year follow-up.

## Materials and methods

The study was approved by the Institutional Review Board of Hospital Universitario Ramón y Cajal (approval number 222-16, November 22nd 2016). All patients received oral and written information about the study and written consent was obtained.

### Trial design

This was a 2-arm, multicentric, triple-blinded, parallel-group, pragmatic, randomized, superiority trial with 12-months follow-up. A total of 71 subjects were randomized either to have nanofractures (microfractures made with a special device that makes narrow [1 mm] and long [9 mm] holes in the bone footprint) performed immediately before arthroscopic supraspinatus tendon repair (Nanofracture) or not (Control).

### Participants

The inclusion criteria were: (1) being 18 years old or older, (2) having a symptomatic supraspinatus tear, (3) that the tear was repairable to the footprint with at least 90% coverage, (3) that the subject was able to understand and consent to participate.

The exclusion criteria were: (1) that the size of the supraspinatus tendon tear, measured during surgery, was larger than 3 cm in either anteroposterior (tear size) or mediolateral (tear retraction) direction, (2) that an preoperative MRI evaluation (performed at most 3 months before the surgery) showed grade 4 fatty infiltration in any rotator cuff tendon according to the criteria defined by Goutalier et al. [9] and adapted for MRI by Fusch et al. [8], and (3) the presence, during surgery or in preoperative MRI, of a subscapularis tear that required repair.

### Interventions

After an initial assessment for eligibility criteria, the surgeon offered the participant to take part in the study. If informed consent was obtained, the participants were brought to the surgical theatre. The arthroscopic rotator cuff repair was performed under general anaesthesia and/or an interscalene nerve block. The posterosuperior cuff tear was debrided and assessed for tear pattern (according to Davidson and Burkhart [7]), size, retraction and reparability. The supraspinatus footprint was debrided of soft tissue and the bone surface was gently decorticated with a burr. A biceps tenotomy/tenodesis, an acromioplasty, or a Mumford procedure were performed if deemed necessary. Once the eligibility criteria were confirmed, randomization to one of the two treatment groups proceeded.

In the control group, a double row or transosseous equivalent repair was performed. In the Nanofracture group the repair was performed likewise, but, immediately prior to placing the first anchor, the nanofractures were performed using a NanoFx Microfracture Instrument (Arthrosurface, Franklin, MA, USA). This device is composed of a long handle with a curved tip and a 1 mm thick needle. The handle tip was placed perpendicular to the bone surface and the needle, driven through the handle, was repeatedly hammered through the bone, making 1 mm thick, 9 mm long holes in it. Starting from the articular edge, the nanofractures were made, 3–5 mm apart, until the lateral border of the debrided footprint was reached, averaging 6–10 holes per square centimetre.

### Outcomes

The following evaluation tools were used:

1. Clinical evaluation was performed preoperatively and 3, 6, and 12 months after surgery using the Constant score and the EQ-5D-3L self-rated general health questionnaire (using both the VAS data and the health index calculated using the Time trade-off system adjusted for the Spanish population according to Badia et al. [3]).
2. Pain evaluation was performed using questions 3–6 of the brief Pain Inventory (that assess verbally pain in a 0–10 discrete scale) preoperatively, one and 3 weeks and 2, 3, 6 and 12 months postoperatively.
3. MRI performed preoperatively and 12 months after surgery.

The primary outcome was the retear rate evaluated in the 12 months postoperative MRI. The continuity of the tendon was assessed using the Sugaya’s classification [17] that classifies the integrity of a repaired tendon in a 5 level scale, grades 1–3 were considered healed and grades 4 and 5 were considered to have failed repair (a retear).

The following secondary outcomes were used: (1) Sugaya’s grade in the 12 months postoperative MRI. (2) Place of the retear of the tendon, either at the footprint or at the musculotendinous junction as described by Trantalis et al. [19]; (3) pain levels assessed during the first year postoperatively, compared to the preoperative levels and between groups; and (4) constant score and EQ-5D-3L scores assessed during the first year postoperatively, compared to the preoperative levels and between groups.

### Randomization and blinding

A random list of numbers was computer generated with an allocation rate of 1:1 using block sizes of 6. A set of 96 sequentially numbered opaque envelopes were prepared by a researcher independent from the study. One block of 6 envelopes was randomly assigned to each team. When any team randomized the first 6 subjects a second set was assigned to that team. Once the surgeon had evaluated arthroscopically the joint, assessed the supraspinatus tear for size and reparability and confirmed that the participant met all the inclusion criteria and none of the exclusion criteria, the envelope was opened by a nurse and shown to the surgeon.

During informed consent, the patients were informed that they would be blinded to the arm of the study they were being assigned to. The surgeon was not blinded to the assignation as he had to perform the nanofractures. Clinical assessment of the participants was done by one surgeon of each team that was unaware of the group assignment. Neither the person assessing the MRI nor the statistician making the statistical analysis were aware of the group assignment.

### Statistical analysis

Sample size calculation was done using the primary outcome: retear rate in the twelve-month postoperative MRI. With an estimated retear rate of 30%, with an alpha error of 0.05 and a power (1 − *β*) of 0.5, a total of 31 participants for each group were required to detect a difference in retear rate between groups of 20%. Accounting for an estimated loss of follow-up of 15% a total number of 71 subjects were included.

All continuous variables were tested for normality using the Kolmogorov–Smirnoff test. Chi-squared test was used to compare dichotomous and qualitative variables. Student’s *T* test was used to compare quantitative variables. The statistical threshold for significance was established at *p* < 0.05. Two logistic regression analyses were performed to assess the possible confounding effect of sex, tear size and retraction, and fatty infiltration at the supraspinatus or infraspinatus muscles in the retear rate or failure to heal at the footprint.

## Results

**Fig. 1:**
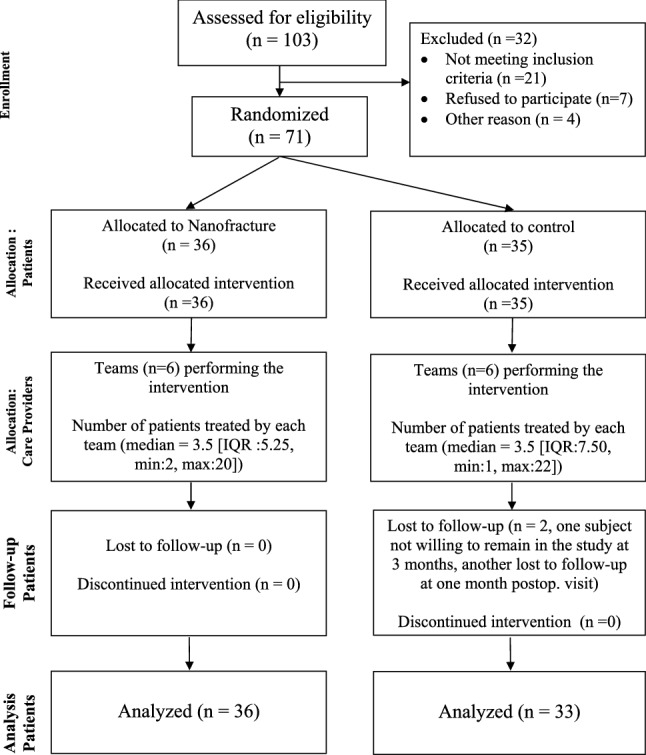
CONSORT flow diagram

The participant flow can be observed in Fig. 1. From a total of 103 subjects assessed for eligibility, 71 were available for randomization. All of these were randomized (36 to nanofracture and 35 to control) and received the allocated intervention. Two subjects (both from the control group) did not fulfil the 1 year follow up: one decided that he wanted to be withdrawn from the study at the 3 month follow-up visit and another did not come to the 1 month visit and was lost to follow-up. Recruitment started on January 2017 and stopped in May 2018.

**Table 1:** Baseline demographic, clinical characteristics and surgical data for each group

	Control	Nanofracture	Significance
*N*	33	36	
Demographic data			
Age	57.8 (10.7)	60.1 (7.88)	n.s.
Sex (male:female)	18:15	14:22	n.s.
BMI	28.6 (6.06)	26.7 (3.56)	n.s.
Tear size and tendon quality			
Side (left:right)	10:23	14:19	n.s.
Type			n.s.
Crescent	25 (75.8%)	26 (72.2%)	
L-shaped	4 (12.1%)	5 (13.9%)	
U-shaped	4 (12.1%)	5 (13.9%)	
Tear size	17.6 (5.49)	20.4 (6.4)	n.s.
Tear retraction	15.7 (6.02)	16.5 (6.78)	n.s.
Fatty infiltration			
SE (0:I:II:III)	19:8:3	19:13:4	n.s.
IE (0:I:II:III)	27:5:1	30:5:1	n.s.
Functional			
Pain levels (BPI)			n.s.
Maximum	7.79 (1.47)	7.94 (1.37)	
Minimum	2.55 (2.09)	3.25 (1.98)	
Mean	5.55 (1.72)	5.83 (1.56)	
Now	5.24 (2.22)	5.81 (2.01)	
Constant score	46.7 (16.3)	42.8 (16.3)	n.s.
Pain	4.21 (3.08)	3.92 (2.83)	n.s.
Functional	9.58 (3.91)	8.72 (3.90)	n.s.
ROM	27.6 (8.80)	25.7 (6.94)	n.s.
Strength	5.29 (6.88)	4.49 (5.73)	n.s.
EQ-5D-3L scores			
Health index (TTO)	0.60 (0.17)	0.59 (0.17)	
Health level (VAS)	61.0 (19.1)	60.7 (20.1)	
Surgical technique			
Doble row:TOE	7:26	9:27	n.s.
Associated procedures			n.s.
Acromioplasty	6 (18.2%)	4 (11.1%)	
Biceps tenotomy	23 (69.7%)	25 (69.4%)	
Biceps tenodesis	1 (3.0%)	1 (2.8%)	
Mumford	2 (6.0%)	3 (8.3%)	
Number of implants	3.5 (1.20)	3.69 (1.06)	n.s.

The number in parenthesis are the standard deviation for quantitative variables and the percentage of the total for each group in quantitative variables

*BMI* body mass index, *SE* supraspinatus, *IE* infraspinatus, *BPI* brief pain inventory, *TTO* time trade-off, *VAS* visual analog score, *TOE* transosseous equivalent, *n.s.* not significant

### Baseline data

The baseline data for each group can be seen in Table 1. There were no significant differences (NS) in any of the demographical data, tear characteristics, muscle fatty infiltration, surgical technique, associated surgical procedures or number of implants used.

Six teams of shoulder surgeons took part in the study including 8 shoulder surgeons working in 8 different institutions across Spain (3 public, 3 private and 2 workers’ compensation hospitals). The six teams recruited a median of 6 participants each (maximum: 42; minimum: 4).

### Outcomes and estimation

There were more retears at one-year follow-up in the Control group (14 retears out of 33 subjects, 42.4%; 95% confidence interval 25.5–60.8%) than in the Nanofracture group (7 out of 36 subjects, (19.4%; 8.2–36.0%; Odds ratio 0.33; 95% confidence interval 0.11–0.96, *p* = 0.042). The absolute reduction of retear risk using nanofractures was 0.23 (0.01–0.44) and the relative reduction of retear risk was 0.46 (0.21–0.99). When assessed according to the Sugaya classification there were no significant differences (3.15 [1.23] in the control group vs. 2.61 [1.10] in the Nanofracture group [n.s].).

When the characteristics of the retear were evaluated (whether the tear developed at the musculotendinous junction or at the footprint), both groups had similar retear rates at the musculotendinous junction: 5/33 (15%; 5–32%) in the control group and 5/36 (13%, 6–30%) in the Nanofracture group (odds ratio: 0.90; 0.24–3.45; n.s.). The nanofracture group had better healing rates at the footprint: 24/33 (73%, 54–87%) healed to the bone in the Control group and 34/36 (94%, 81–99%) in the nanofracture group (odds ratio 0.16, 95% confidence interval 0.03–0.79, *p* = 0.025). The absolute reduction of the retear risk at the footprint using nanofractures was 0.21 (0.05–0.38) and the relative reduction of retear risk was 0.27 (0.05–0.87).

Pain levels evolved favourably during the twelve-month follow up (Table 2). Pain levels improved all along the follow-up period. The total constant scores of both groups improved significantly compared to the preoperative values at the 6-months and 12-months postoperative evaluations (*p* < 0.001, Table 3). All subscales for the constant score evolved in a similar pattern. For both parts of the EQ-5D-3L (the health index score and the VAS) there were significant improvements compared to the preoperative values at the six-months and one-year postoperative evaluations (*p* < 0.01, Table 4). There were no differences in pain levels, constant scores, or quality of life between both groups (nanofracture vs. control) at any time.

**Table 2:** Pain levels of both groups during the first year after surgery

Control group	Preoperative	6 months	1 year
Maximum pain	7.8 (1.4)	*3.7 (2.4)*	*1.9 (2.5)*
Mean pain	2.6 (2.1)	*0.8 (1.8)*	*0.7 (1.6)*
Minimum pain	5.6 (1.7)	*1.9 (2.2)*	*1.3 (2.1)*
Pain Now	5.2 (2.2)	*1.4 (2.2)*	*1 (1.8)*

Nanofracture group	Preoperative	6 months	1 year
Maximum pain	7.9 (1.4)	*2.4 (2.3)*	*1.6 (2.4)*
Minimum pain	3.2 (2.0)	*0.4 (0.9)*	*0.2 (0.7)*
Mean pain	5.8 (1.6)	*1.3 (1.6)*	*0.9 (1.6)*
Pain Now	5.8 (2.0)	*0.7 (1.3)*	*0.5 (1.6)*

The subjects answered four questions of the brief Pain Inventory (that assess verbally pain in a 0 to 10 discrete scale) preoperatively. 1 and 3 weeks and 2, 3, 6 and 12 months postoperatively. Numbers in brackets are the SD. Pain improved in both groups significatively but did not differ between groups. There were significant improvements in all variables (* p* < 0.05) when comparing the preoperative values with either the 6 months or one year values

**Table 3:** Total constant score (and subscales) of both groups preoperatively, 6 and 12 months postoperatively

Control group	Preoperative	6 months	1 year
Pain	4.2 (3.1)	*9.7 (3.7)*	*12.1 (4)*
ADL	9.6 (3.9)	*15.4 (5.3)*	*17.2 (4.9)*
ROM	27.6 (8.8)	*34.8 (9.8)*	*35.5 (9.8)*
Strength	5.3 (6.9)	*8.9 (7.4)*	*12.1 (7.8)*
Total constant score	46.7 (16.3)	*68.3 (21.9)*	*76.2 (24.1)*

Nanofracture group	Preoperative	6 months	1 year
Pain	3.9 (2.8)	*11.2 (3.5)*	*13.2 (2.7)*
ADL	8.7 (3.9)	*16.7 (3.5)*	*18.3 (2.4)*
ROM	25.7 (6.9)	*35.3 (5.6)*	*38.3 (2.5)*
Strength	4.5 (5.7)	*10.9 (6.9)*	*14.8 (7)*
Total constant score	42.8 (14.7)	*74.1(15)*	*84.6 (11.8)*

The total constant score and all the subscales improved in both groups significatively. Starting at the six-months follow-up visit but did not differ between groups. Numbers in brackets are the SD. There were significant improvements in all variables (*p* < 0.05) when comparing the preoperative values with either the 6 months or one year values

**Table 4:** Outcomes of the EQ- of both groups preoperatively, at 6 and 12 months postoperatively

Control group	Preoperative	6 months	1 year
Health index (TTO)	0.6 (0.17)	*0.86 (0.21)*	*0.89 (0.19)*
VAS	61 (20)	*72 (17)*	*79 (16)*

Nanofracture Group	Preoperative	6 months	1 year
Health index (TTO)	0.59 (0.17)	*0.85 (0.13)*	*0.88 (0.09)*
VAS	61 (19)	*72 (20)*	*78 (21)*

The health index (calculated by the time trade-off system) and the VAS data are presented. Both the health index and the VAS score improved in both groups significatively in all postoperative measures. But did not differ between treatment groups. Numbers in brackets are the SD. There were significant improvements in all variables (*p* < 0.05) when comparing the preoperative values with either the 6 months or one year values

The logistic regression analysis confirmed the previous findings. After adjusting for age, sex, tear size and muscle fatty infiltration the Odds ratio for the effect of nanofractures on retear rates was 0.15 (95% confidence interval 0.03–0.63, *p* = 0.009). For failure to heal to the bone the odds ratio was 0.09 (95% confidence interval 0.01–0.64, *p* = 0.016).

### Harms

No significant complications developed in the Control group during the study. Three complications were noted in the nanofracture group (n.s.). In one case a trabecular fracture developed during implant insertion that required a change in position of the implant but did not preclude a satisfactory repair. This subject eventually developed a re-rupture at the musculotendinous junction with a final constant score of 86. Two participants presented with swelling and pain at the shoulder at the one-week follow-up and the suspicion of an infection was established. In the first case the problem was mild and a 1-week course of oral antibiotics solved the symptoms, the tendon healed successfully and the final constant score was 87. In the second case, a deep infection was suspected, and an arthroscopic lavage of the joint was performed followed by a 10-day course of intravenous antibiotics. Intraoperative cultures were negative, the subject improved, the tendon healed successfully, and the final constant score was 95.

## Discussion

The main finding of this study is that adding nanofractures to the footprint of a mid-size suprapinatus tear repaired with a double row or transosseous equivalent technique increases the chances of healing of the tendon when assessed with MRI at 12-months follow-up. These increased healing chances seem to be due to increased healing at the footprint, but the retear rate at the musculotendinous junction does not vary.

Different previous RCT have looked for evidence of the efficacy of bone marrow stimulation with microfractures. Milano et al. [14] and Osti et al. [15] performed two randomized controlled trials to evaluate the efficacy of microfractures (done with an arthroscopic awl that created 1.5–2 mm wide, 3–5 mm deep, holes) at the footprint during rotator cuff repair but did not find differences in structural healing (evaluated with MRI) or functional outcomes at 1 year. Jo et al. [12], in a lower quality cohort study, did find differences in the retear rate when performing microfractures (with a 2 mm × 10 mm bone punch): they found retear rates similar to our study with 22% retear rate in the microfracture group and 45% in the control group. Taiguchi et al. [18] in a retrospective cohort study also found differences with retear rates in the microfracture group of 9% and 24% in the control group, the microfractures were performed with an 3 mm-arthroscopic awl. To summarize this data, Arjawat et al. [2] made a meta-analysis of these four studies and found that microfractures decreased in half the chances of finding a re-tear at 1 year (odds ratio, 0.42; 95% confidence interval: 0.25–0.73; *p* = 0.002). Thus, the results of this study seem to fall in line with those found by other authors.

One novel information that this study provided is that the retear rate at the musculotendinous junction did not seem to be affected by the nanofractures, but it did affect the chances of healing at the footprint. This makes sense, as nanofractures should help the tendon-to-bone healing, but cannot avoid medial tears.

The functional outcomes and quality of life measures improved significantly after surgery in both groups but no differences between groups were observed despite the differences in retear rates. This is partly expected as the association between anatomical outcomes and clinical outcomes after rotator cuff repair is often weak [16]. For example, in the recent meta-analysis by Arjawat et al. [2], no differences in clinical outcomes were observed.

In this study, three complications developed in the Nanofracture group. None of these affected the clinical outcome. In the case where a retear developed after a trabecular fracture during implant insertion, the retear developed at the musculotendinous junction, not at the “fractured” footprint. Two other cases developed postoperative swelling with negative cultures. These might be related to some degree of increased early postoperative inflammation of the shoulder due to the increased insult to the bone during microfracturing.

There are some limitations to this study. A first limitation is that eight different surgeons performed the surgical procedures, and these was indeed some variability of the techniques uses, this could add relevant variability to the study. Despite this, an adequate choice of comparator, good blinding, similar expertise of the surgeons, strict randomization and a well-powered study allowed for the valid result to be obtained. Another limitation is that a single team of surgeons performed most of the cases (42 of 71 participants assessed for outcomes), this could limit the generalizability of the results. Despite this, the team included three different surgeons that worked in a large University hospital and a private practice centre, so the diversity of the subjects included was still high. The last limitation is that, when performing sample size calculation, a power of only 0.5 was selected to limit the sample size to something feasible (using an optimal 0.90 power would require over 200 participants).

The results obtained here can be generalized to the general population with midsize supraspinatus tears. The varied origin of the subjects included (coming form 8 centers in 6 cities around Spain and including private patients, national health service patients and worker compensation patients) assures that the results could be extrapolated to the general population.

## Conclusions

Adding nanofractures at the footprint during an isolated supraspinatus repair lowers in half the retear rate at 12-months follow-up. This is due to improved healing at the footprint.

## Protocol

A full copy (in Spanish) of the IRB-approved protocol can be seen in Online Appendix 1.

A full copy (in Spanish) of the CRD can be seen in Online Appendix 2.

## Electronic supplementary material

Below is the link to the electronic supplementary material.

Supplementary material 1 (PDF 731 kb)

Supplementary material 2 (PDF 1364 kb)
