# Correction to: Footprint preparation with nanofractures in a supraspinatus repair cuts in half the retear rate at 1-year follow-up. A randomized controlled trial

**DOI:** 10.1007/s00167-020-06118-x

**Published:** 2020-06-27

**Authors:** Miguel Angel Ruiz Ibán, Eduardo Sanchez Alepuz, Jorge Diaz Heredia, Abdul-ilah Hachem, Leon Ezagüi Bentolila, Angel Calvo, Carlos Verdú, Ignacio de Rus Aznar, Francesc Soler Romagosa

**Affiliations:** 1 Unidad de Hombro y Codo, Hospital Universitario Ramón y Cajal, Cta Colmenar km 9,100, 28046 Madrid, Spain; 2 Hospital IMED Valencia, Valencia, Spain; 3 Head of the Shoulder Unit, Hospital Universitario de Bellvitge, Barcelona, Spain; 4 Hospital Egarsat, Barcelona, Spain; 5 Arthrosport, Zaragoza, Spain; 6 Unidad de Hombro y Codo, Hospital General Universitario de Elche, Elche, Alicante Spain; 7 Traumadvance, Terrassa, Barcelona, Spain

## Correction to: Knee Surgery, Sports Traumatology, Arthroscopy https://doi.org/10.1007/s00167-020-06073-7

The article Footprint preparation with nanofractures in a supraspinatus repair cuts in half the retear rate at 1-year follow-up. A randomized controlled trial, written by Miguel Angel Ruiz Ibán, Eduardo Sanchez Alepuz, Jorge Diaz Heredia, Abdul-ilah Hachem, Leon Ezagüi Bentolila, Angel Calvo, Carlos Verdú, Ignacio de Rus Aznar, Francesc Soler Romagosa, was originally published electronically on the publisher’s internet portal on 01 June, 2020 without open access. With the author(s)’ decision to opt for Open Choice the copyright of the article changed on 27 June, 2020 to © The Author(s) 2020 and the article is forthwith distributed under a Creative Commons Attribution 4.0 International License (https://creativecommons.org/licenses/by/4.0/), which permits use, sharing, adaptation, distribution and reproduction in any medium or format, as long as you give appropriate credit to the original author(s) and the source, provide a link to the Creative Commons licence, and indicate if changes were made.

The original article has been corrected.

