# Supplementary material for: Footprint preparation with nanofractures in a supraspinatus repair cuts in half the retear rate at 1-year follow-up. A randomized controlled trial
Source: Knee Surg Sports Traumatol Arthrosc. 2020 Jun 1;29(7):2249–56. doi: 10.1007/s00167-020-06073-7 (PMC8225541; doi:10.1007/s00167-020-06073-7)
Supplement: Supplementary file 1 — Supplementary material 1 (PDF 731 kb) [file 167_2020_6073_MOESM1_ESM.pdf]

PROTOCOLO

CÓDIGO: (MICROMANGUITO01)

Versión: 03, 21 de noviembre de 2016

APROBADO POR EL CEIC DEL HOSPITAL UNIVESITARIO

RAMON Y CAJAL EL 22 DE NOVIEMBRE DE 2016

TITULO:

EFECTO EN EL ÍNDICE DE CICATRIZACIÓN DE LA REALIZACIÓN DE NANOFRACTURAS EN LA HUELLA OSEA DE REPARACIÓN DE ROTURAS DEL TENDÓN DEL SUPRAESPINOSO DEL HOMBRO

DECLARACIÓN DEL INVESTIGADOR

- Acepto Asumir la responsabilidad de que se lleve a cabo correctamente el estudio en este Centro
- Acepto realizar el estudio de conformidad con el presente protocolo **Versión 03**. de 21 de noviembre de 2016
- No introduciré ningún cambio en el protocolo sin el consentimiento previo del promotor y la autorización por escrito del Comité de ética de la investigación con medicamentos (CEIm), excepto cuando sea preciso para contrarrestar cualquier peligro inminente que puedan correr los pacientes o en cuanto se refiera a los aspectos administrativos del estudio (y cuando esté permitido por la normativa legal vigente a este efecto
- Conozco y cumpliré las normas de buena práctica clínica (BPC) y los demás requisitos legales pertinentes

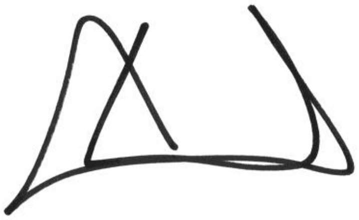

Fdo: MIGUEL ANGEL RUIZ IBAN Fecha: 21/11/2016

Investigador principal del estudio

ÍNDICE

DECLARACIÓN DEL INVESTIGADOR *2*

ÍNDICE *3*

1. RESUMEN *7*

2. INFORMACIÓN GENERAL *11*

2.1. Identificación del ensayo 11

2.2. Tipo de ensayo clínico 11

2.3. Datos relativos al promotor 11

MIGUEL ANGEL RUIZ IBAN 11

2.4. Identificación del monitor 11

2.6. Expertos médicos del promotor: 12

2.7. Investigador principal y colaboradores: 12

2.8. Centro(s) de investigación: 13

2.9. Médico responsable de las decisiones médicas: 13

2.10. Laboratorios clínicos: 13

3. JUSTIFICACIÓN Y OBJETIVOS *14*

3.1. Pertinencia del ensayo clínico. 14

3.2. Descripción del producto en estudio. 17

3.3. Descripción de la población a estudiar. 18

3.4. Objetivo principal. 18

3.5. Objetivos secundarios. 18

4. TIPO DE ENSAYO Y DISEÑO *18*

4.1. Fase del ensayo clínico. 18
4.2. Variables principales y secundarias. 19
4.3. Diseño. 20
Ensayo clínico prospectivo, aleatorizado, de grupos paralelos y ciego para el sujeto y para el evaluador 20
4.4. Medidas para evitar sesgos. 20
4.5. Tratamiento. 20
4.6. Duración. 21
4.7. Criterios de finalización. 21
4.8. Contabilización del medicamento. 21
4.9. Códigos de aleatorización/ciego. 21
4.10. Datos fuente. 22
4.11. Definición de final de ensayo clínico. 23
5. SELECCIÓN Y RETIRADA DE SUJETOS ***23***
5.1. Criterios de inclusión 23
5.2. Criterios de exclusión. 24
5.3. Número de sujetos previsto y justificación. 24
5.4. Criterios de retirada y análisis. 25
5.5. Duración aproximada del periodo de reclutamiento. 25
6. DESCRIPCIÓN DEL TRATAMIENTO ***25***
6.1. Definición del tratamiento. 25
6.2. Tratamientos concomitantes. 25
6.3. Cumplimiento. 26
7. EVALUACIÓN DE LA EFICACIA ***26***
7.1. Criterios de valoración de eficacia. 26
7.2. Desarrollo del estudio. 27
7.3. Descripción de los métodos para la valoración de la eficacia. 28
7.3.1. Determinaciones de laboratorio. 29

8. SEGURIDAD ***29***

8.1. DEFINICIONES 29

8.1.1. Acontecimiento Adverso, AA (AE, adverse event) 29
8.1.2. Efecto Adverso del Producto, EAP (ADE, adverse device effect) 29
8.1.3. Acontecimiento Adverso Grave, AAG (SAE, serious adverse event) 29
8.1.4. Efecto Adverso Grave del Producto en investigación, EAGP (SADE, serious adverse device effect)30
8.1.5. Efecto adverso grave inesperado del producto, EAGIP (USADE, unanticipated Serious Adverse Device Effect) 30
8.1.6. Deficiencia del producto 31

8.2. Informacion de Seguridad de Referencia 31

8.3. Intensidad 31

8.4. Causalidad 31

8.5. Procedimiento de recogida de los Acontecimientos Adversos 32

8.6. Notificacion de Acontecimientos Adversos 33

8.7. Notificación Expeditiva de otra información de seguridad relevante 34

8.8. Embarazos 34

9. ESTADÍSTICA ***35***

9.1. Cálculo del tamaño muestral. 35

9.2. Métodos estadísticos. 35

9.3. Criterios para la finalización del ensayo. 35

9.4. Pérdida de datos. 36

9.5. Desviaciones del plan estadístico. 36

9.6. Selección de sujetos para el análisis. 36

10. ACCESO A LOS DATOS/DOCUMENTOS FUENTE ***37***

11. CONTROL Y GARANTÍA DE CALIDAD ***38***

12. ASPECTOS ÉTICOS ***39***

12.1. **Normas generales y particulares para los investigadores** 39
12.2. **Consentimiento informado** 39
12.3. **Dispositivos de seguridad y confidencialidad** 39
12.4. **Contenido del presupuesto del ensayo** 39
13. CONSIDERACIONES PRÁCTICAS ***40***
13.1. **Responsabilidades de los participantes del ensayo clínico** 40
13.2. **Desviaciones del protocolo** 41
13.3. **Archivo de la documentación** 41
13.4. **Enmiendas al protocolo** 42
13.5. **Aceptación del investigador** 42
13.6. **Condiciones de publicación** 42
14. APÉNDICES ***43***

1. RESUMEN

0. Tipo de solicitud

Ensayo clínico postautorización para un producto sanitario ya aprobado en la UE.

1. Promotor

MIGUEL ANGEL RUIZ IBAN

2.Título del ensayo clínico

EFECTO EN EL ÍNDICE DE CICATRIZACIÓN DE LA REALIZACIÓN DE NANOFRACTURAS EN LA HUELLA OSEA DE REPARACIÓN DE ROTURAS DEL TENDÓN DEL SUPRAESPINOSO DEL HOMBRO

3. Código del protocolo: MICROMANGUITO01

4. Investigador principal y dirección de su centro de trabajo

MIGUEL ANGEL RUIZ IBAN

Servicio De Traumatología Y Cirugía Ortopédica

Hospital Ramón y Cajal

Ctra. de Colmenar Km 9,100

28034 Madrid.

Tel y Fax: 913368208 655668479

e-mail: drmri@hotmail.com

5. Centros en los que se prevé realizar el ensayo

Hospital Ramón y Cajal (Madrid)

Clínica Arthrosport (Valencia)

Hospital Clínic (Barcelona)

Unión de Mutuas (Valencia)

Egarsat (Terrassa)

Hospital Rey Juan Carlos (Alcorcón)

Hospital de la Santa Creu i Sant Pau (Barcelona)

6. Comité de ética de la Investigación que evalúa el ensayo

CEIm Hospital Universitario Ramón y Cajal.

7. Nombre y calificación de la persona responsable de la monitorización

Jorge Díaz Heredia

Facultativo del Servicio de Cirugía Ortopédica y Traumatología del Hospital Universitario Ramón y Cajal

8. Producto sanitario a estudio y control

Producto sanitario en estudio:

NanoFx Microfracture Instrument. 15 degree Microfracture Instrument. Modelo: 5500-1020. ARthrosurface.

Producto control:

Ninguno

9. Fase del ensayo clínico

Ensayo clínico en fase IV, con producto sanitario con marcado CE en las condiciones autorizadas para su uso.

10. Objetivo principal

Definir si el uso del **NanoFx Microfracture Instrument para la realización de microfracturas** en la huella de reinserción tendinosa mejora la tasa de integridad del tendón supraespinoso tras ser suturado a la huella ósea de inserción mediante evaluación con resonancia magnética frente a la técnica estándar de reparación del tendón sin realización de microfracturas.

11. Diseño

Ensayo clínico prospectivo aleatorizado, grupos paralelos, ciego para el evaluador y para el paciente de 2 años de duración

12. Enfermedad o trastorno en estudio

Roturas del tendón del supraespinoso del hombro

13. Variable principal de valoración

Integridad del tendón supraespinoso reparado en estudio de resonancia magnética al año de la intervención.

14. Población en estudio y número total de pacientes

Pacientes con roturas aisladas del supraespinoso

60 pacientes (30 en cada grupo)

15. Duración del tratamiento

El producto sanitario se utiliza una sola vez durante el procedimiento quirúrgico. Los pacientes son seguidos clínica y radiológicamente durante un año tras la intervención

16. Calendario y fecha prevista de realización

Tras la aprobación por el Comité Ético del Hospital Universitario Ramón y Cajal:

- Inclusión de pacientes: 12 meses
- Duración del tratamiento y seguimiento: 12 meses de seguimiento

En total se estima terminar el estudio 24 meses después de la aprobación.

2. *INFORMACIÓN GENERAL*

2.1. Identificación del ensayo

Título: EFECTO EN EL ÍNDICE DE CICATRIZACIÓN DE LA REALIZACIÓN DE NANOFRACTURAS EN LA HUELLA OSEA DE REPARACIÓN DE ROTURAS DEL TENDÓN DEL SUPRAESPINOSO DEL HOMBRO

Código del protocolo: MICROMANGUITO01
1 de septiembre de 2016

2.2. Tipo de ensayo clínico

Ensayo clínico aleatorizado ciego para paciente y evaluador con un dispositivo aprobado y con marcado CE

2.3. Datos relativos al promotor

MIGUEL ANGEL RUIZ IBAN

Servicio De Traumatología Y Cirugía Ortopédica

Hospital Universitario Ramón y Cajal

Ctra. de Colmenar Km 9,100

28034 Madrid.

Tel y Fax: 913368208 655668479

e-mail: drmri@hotmail.com

2.4. Identificación del monitor

Jorge Díaz Heredia

Facultativo del Servicio de Cirugía Ortopédica y Traumatología del Hospital Universitario Ramón y Cajal

2.6. Expertos médicos del promotor:

MIGUEL ANGEL RUIZ IBAN

Servicio De Traumatología Y Cirugía Ortopédica

Hospital Ramón y Cajal

Ctra. de Colmenar Km 9,100

28034 Madrid.

Tel y Fax: 913368208 655668479

e-mail: drmri@hotmail.com

2.7. Investigador principal y colaboradores:

Investigador Principal:

MIGUEL ANGEL RUIZ IBAN

Servicio De Traumatología Y Cirugía Ortopédica

Hospital Ramón y Cajal

Ctra. de Colmenar Km 9,100

28034 Madrid.

Tel y Fax: 913368208 655668479

e-mail: drmri@hotmail.com

Investigadores Principales Colaboradores em cada centro:

Dr. Angel Calvo. Clínica Arthrosport (Valencia)

Dr. Sergi Sastre. Hospital Clínic (Barcelona)

Dr. Eduardo Sánchez Alepuz. Unión de Mútuas (Valencia)

Dr. Francesc Soler Romagosa. Egarsat (Terrassa)

Dr. Juan Antonio Torres Rubio (Alcorcón)
Dr Juan Sarasquete Reiriz

2.8. Centro(s) de investigación:

Hospital Ramón y Cajal

Clínica Arthrosport (Valencia)

Hospital Clínic (Barcelona)

Unión de Mútuas (Valencia)

Egarsat (Terrassa)

Hospital Rey Juan Carlos (Alcorcón)
Hospital de la Santa Creu i Sant Pau (Barcelona)

2.9. Médico responsable de las decisiones médicas:

El investigador principal

2.10. Laboratorios clínicos:

No son necesarios en el ensayo

3. JUSTIFICACIÓN Y OBJETIVOS

3.1. Pertinencia del ensayo clínico.

La rotura de la cofia rotadora es una de las patologías más frecuentes en la articulación del hombro. El número de pacientes afectados de rotura de la cofia rotadora aumenta con la edad, y en los casos que se manifiesta clínicamente es causa de dolor e impotencia funcional (1,2,3). Cuando aparece la sintomatología clínica el tratamiento de elección es el quirúrgico, realizando una reinserción del cabo tendinoso en su lecho óseo original en la parte superior de la tuberosidad mayor del húmero. Esta intervención se puede realizar por técnica abierta o por técnica artroscópica, siendo esta última cada vez más frecuente (4).

La tasa de éxito de la curación de la rotura de la cofia rotadora después del tratamiento quirúrgico oscila según las series entre el 30% y el 80% (5), siendo el porcentaje inferior para aquellas roturas clasificadas como grandes o masivas así como en las que ya hay signos de degeneración del tendón. En estos tendones, debido a su pobre vascularización intrínseca, hay una insuficiente migración de células implicadas en el proceso de cicatrización de la rotura, lo que impide el éxito de la misma (6,7).

Las células implicadas en el proceso de cicatrización tendinoso provienen del propio tendón (células intrínsecas), de la sinovial articular y las células pluripotenciales provenientes de la medular ósea (células extrínsecas) (8,9,10,11,12,13). Estas últimas tienen la capacidad de diferenciarse en tejido tendinoso (14) y son vitales en el proceso de reparación tendinosa.

Kida et al demostró un mayor índice de cicatrización tendinosa en las suturas de la cofia rotadora en ratas donde aportaba células mesenquimales a nivel de la sutura entre el hueso y el tendón (15).

Diferentes estudios han demostrado que la realización de microfracturas en el lecho óseo incrementa la presencia de células pluripotenciales provenientes de la medula ósea a nivel de la unión entre el hueso y el tendón (16,17).

Las nanofracturas (microperforaciones de menor diámetro y mayor profundidad) realizadas con el **NanoFx Microfracture Instrument** permiten un acceso más profundo a nivel de la medular ósea

(9mm de profundidad frente a 3mm de las microfracturas), con una mayor conservación del remanente óseo (1mm de anchura frente a 2mm de las microfracturas), por lo que se facilita el acceso de las células pluripotenciales a nivel de la unión hueso-tendón (18).

Milano et al en un estudio preliminar demostró en un estudio prospectivo randomizado un mayor índice de curación en las reinserciones de la cofia rotadora en las que se había realizado microfracturas con el **NanoFx Microfracture Instrument** en el lecho de inserción del tendón previo a su sutura (19).

Estos estudios y otros han tenido un impacto relativo en la práctica clínica habitual ya que no se encuentran refrendados por ECA de calidad. De esta manera los cirujanos toman actitudes muy distintas a la hora de preparar el lecho óseo sobre le que se repara el tendón: desde no hacer nada específico sobre este, sin apenas retirar los restos de tendón existentes a hacer resecciones de la cortical superficial del hueso para exponer el hueso esponjoso subyacente asociando o no perforaciones a ese nivel. No existe consenso entonces sobre cual es la mejor alternativa y la decisión de aplicar uno u otro procedimiento está en función de la preferencia de cada cirujano.

El objetivo del estudio es evaluar si la realización de microfracturas con este producto a nivel de la tuberosidad mayor humeral en el momento de la reparación tendinosa tiene efecto en el éxito de dicha reparación. El producto se utilizará en el momento de la intervención quirúrgica de reparación del manguito y se usará de acuerdo a las instrucciones del fabricante.

El estudio no supone una desviación de la practica clínica habitual en estos sujetos donde se realiza sistemáticamente una preparación del lecho óseo para el implante del tendón por lo que el uso de este producto no lleva asociados riesgos adicionales. En el único estudio previo que se utilizó dicho producto no se apreciaron complicaciones ni efectos adversos relacionados con el uso de dicho producto(19).

REFERENCIAS BIBLIOGRÁFICAS:

1. Burbank KM, Stevenson JH, Czarnecki GR, Dorfman J. Chronic shoulder pain: part Evaluation and diagnosis. Am Fam Physician 2008;77:453-60.
2. Hawkins RJ, Kennedy JC. Impingement syndrome in athletes. Am J Sports Med 1980;8:151-8.
3. Neer CS II. Anterior acromioplasty for the chronic impingement syndrome

in the shoulder: a preliminary report. J Bone Joint Surg Am 1972;54:41-50.
4. Sugaya H, Maeda K, Matsuki K, Moriishi J. Repair integrity and functional outcome after arthroscopic double-row rotator cuff repair. A prospective outcome study. J Bone Joint Surg Am 2007;89:953-60. doi:10.2106/JBJS.F.00512.
5. Duquin TR, Buyea C, Bisson LJ. Which method of rotator cuff repair leads to the highest rate of structural healing? A systematic review. Am J Sports Med 2010;38:835-841.
6. Benjamin M, Ralphs JR. Tendons and ligaments–an overview. Histol Histopathol 1997;12:1135-44.
7. Funakoshi T, Iwasaki N, Kamishima T, Nishida M, Ito Y, Kondo M, et al. In vivo visualization of vascular patterns of rotator cuff tears using contrast-enhanced ultrasound. Am J Sports Med 2010;38:2464- 71. doi:10.1177/0363546510375536
8. Bucala R, Spiegel LA, Chesney J, Hogan M, Cerami A. Circulating fibrocytes define a new leukocyte subpopulation that mediates tissue repair. Mol Med 1994;1:71-81.
9. Chesney J, Bucala R. Peripheral blood fibrocytes: novel fibroblast-like cells that present antigen and mediate tissue repair. Biochem Soc Trans 1997;25:520-4.
10. Gulotta LV, Kovacevic D, Ehteshami JR, Dagher E, Packer JD, Rodeo SA. Application of bone marrow-derived mesenchymal stem cells in a rotator cuff repair model. Am J Sports Med 2009;37:2126- 33. doi:10.1177/0363546509339582
11. Ju YJ, Muneta T, Yoshimura H, Koga H, Sekiya I. Synovial mesenchymal stem cells accelerate early remodeling of tendon-bone healing. Cell Tissue Res 2008;332:469-78. doi:10.1007/s00441-008-0610-z
12. Lundborg G. Experimental flexor tendon healing without adhesion formation–a new concept of tendon nutrition and intrinsic healing mechanisms. A preliminary report. Hand 1976;8:235-8.
13. Pittenger MF, Mackay AM, Beck SC, Jaiswal RK, Douglas R, Mosca JD, et al. Multilineage potential of adult human mesenchymal stem cells. Science 1999;284:143-7.
14. Caplan AI. Why are MSCs therapeutic? New data: new insight. J Pathol 2009;217:318-24. doi:10.1002/path.2469
15. Kida Y, Morihara T, Kubo T. Bone Marrow–Derived Cells frm the Footprint Infiltrate into the Repaired Rotator Cuff. J Shoulder Elbow Surg 2013; 22, 197-205
16. Snyder SJ, Burns J. Rotator cuff healing and the bone marrow “crimson duvet” from clinical observations to science. Tech Shoulder Elbow Surg 2009;10:130-137.
17. Taniguchi N, Suenaga N, Oizumi N. Bone Marrow Stimulation at the Footprint of Arthroscopic Surface Holding Repair Advances Cuff Repair Integrity. J Shoulder Elbow Surg. 2015;24(6): 860-866.

18. Benthien J and Behrens P. Reviewing subchondral cartilage surgery: considerations for standardized, deep marrow stimulation. A Technical Note. Int Orthop 2013 Nov;37(11):2139-45.
19. Milano G, Saccomano MF, Careri S, Taccardo G, De Vitis R, Fabricciani C. Efficacy of Marrow-Stimulating Technique in Arthroscopic Rotator Cuff Repair: A Prospective Randomized Study. Arthroscopy: The Journal of Arthroscopic and Related Surgery, Vol 29, No 5 (May), 2013

3.2. Descripción del producto en estudio.

El **NanoFx Microfracture Instrument** es un producto diseñado para la realización de microfracturas en lechos óseos de lesiones condrales y otras localizaciones. El producto consta de una pieza de mano, un accesorio para extracción y una aguja-broca de 1mm de diámetro con un tope proximal.

El sistema se introduce en el espacio subacromial del sujeto a través de uno de los portales artroscópicos tras hacer la preparación estándar de la huella de inserción del supraespinoso (resección de los restos tendinosos, exposición del hueso y regularización de éste). La pieza de mano se coloca sobre el lecho óseo y se impacta la aguja-broca en le interior del hueso. Se retira con ayuda del accesorio para extracción, se recoloca y se realizan las perforaciones necesarias según la extensión de la huella.

El sistema se ha utilizado de manera ocasional en el Hospital Universitario Ramón y Cajal en pacientes con roturas del manguito rotador. Los casos iniciales no han presentado complicaciones relacionadas con el uso del implante, aunque su beneficio clínico no ha sido evidente por la dificultad de la valoración de los resultados en estos sujetos. De esta manera en la actualidad se utiliza ocasionalmente por algunos de los cirujanos de hombro del centro y no por otros.

El producto se encuentra disponible y en uso en todos los centros que van a participar en el estudio, incluido el Hospital Universitario Ramón y Cajal. La empresa proveedora se compromete a ceder, sin coste, los productos que se utilizarán durante este estudio.

El producto es de manejo sencillo y todos los investigadores de los distintos centros tiene experiencia en su uso en la indicación seleccionada y en cirugía de rodilla.

3.3. Descripción de la población a estudiar.

En este estudio se incluirán pacientes que presenten roturas aisladas del tendón supraespinosos sintomáticas y que no respondan al tratamiento conservador. Se excluirán aquellos pacientes en los que se considera que el pronóstico de la reparación es malo (que presenten retracción de los cabos tendinosos o atrofia del músculo, confirmadas por RM y evaluación intraoperatoria) o presenten lesiones asociadas de otros tendones que pudiesen interferir en la evaluación funcional.

3.4. Objetivo principal.

Definir si el uso del **NanoFx Microfracture Instrument para la realización de microfracturas** en la huella de reinserción tendinosa mejora la tasa de integridad del tendón supraespinoso tras ser suturado a la huella ósea de inserción mediante evaluación con resonancia magnética frente a la técnica estándar de reparación del tendón sin realización de microfracturas.

3.5. Objetivos secundarios.

Definir si el uso del **NanoFx Microfracture Instrument** en la huella mejora los resultados funcionales y de dolor postoperatorio mediante evaluación con distintas escalas clínicas frente a la técnica estandar de reparación del tendón sin realización de microfracturas.

4. TIPO DE ENSAYO Y DISEÑO

4.1. Fase del ensayo clínico.

Ensayo clínico aleatorizado, fase IV postautorización, con un dispositivo aprobado y con marcado CE

4.2. Variables principales y secundarias.

Variable principal:

Integridad del tendón reparado evaluado en resonancia magnética nuclear realizada al año de la intervención del tendón del supraespinoso. Definido como una de las siguientes alternativas: **0** Normal , **1** Tendinosis (alteración de señal, sin signos de rotura), **2** Rotura de espesor parcial (cara bursal / cara articular), **3** Rotura de total.

Adicionalmente se realizará un análisis topográfico del tamaño de la rotura en dicha resonancia magnética evaluando el tamaño total de la huella de inserción del supraespinoso y la cantidad de esta (definida en porcentaje sobre la huella total) que queda descubierta (si ntejido tendinoso superpuesto) al año de la reparación.

Variables secundarias:

Niveles de dolor medidos con las preguntas las preguntas 3 a 6 del Brief Pain Inventory (apéndice A).

Resultado funcional medido con la Escala de Constant-Murley (apéndice B), así como sus subdivisiones en dolor, función y movilidad.

Impacto en la calidad de vida del paciente medico con la escala EQ-5D-3L (apéndice C).

Aparición de complicaciones relacionadas con el uso del producto o no en el periodo intra y postoperatorio (infección, rigidez, fractura del troquíter)

4.3. Diseño.

Ensayo clínico prospectivo, aleatorizado, de grupos paralelos y ciego para el sujeto y para el evaluador

4.4. Medidas para evitar sesgos.

Dado que la aleatorización se produce durante la intervención y tras la selección inicial del paciente en consulta, se evitan sesgos de selección.

Los sesgos de evaluación se controlarán mediante el cegado para el paciente y para el evaluador responsable de la evaluación clínica y funcional así como para el evaluador de la resonancia magnética. Dadas las características del producto y de las microfracturas realizadas con él, no existen indicios clínicos o radiológicos que pudiesen sugerir a los evaluadores independientes a que grupo pertenece el paciente. Dado que la evaluación con resonancia magnética se realiza al año de la intervención no habrá evidencia de la realización de microfracturas en el grupo de tratamiento.

4.5. Tratamiento.

El producto se utilizará durante la intervención quirúrgica. Se identificará la lesión del tendón y se realizará una limpieza del tendón lesionado midiendo la extensión de la lesión tendinosa con ayuda de un palpador graduado para confirmar que cumple los criterios de inclusión respecto a tamaño y naturaleza de la lesión.

En todos los sujetos se resecarán los restos de manguito rotador adheridos a la huella ósea de inserción del tendón. Asimismo se resecará con fresa motorizada los primeros 1-2mm de la superficie de hueso cortical.

En ese momento se procederá a la apertura del sobre con el tratamiento asignado y a la asignación del paciente al grupo de tratamiento elegido:

1.-En el grupo control no se realizará ningún gesto adicional sobre el hueso.

2.-En el grupo de tratamiento se utilizará el producto para realizar los orificios en el hueso. según las instrucciones del fabricante, realizando múltiples orificios de 1mm de diámetro en el hueso, separados 2-3 mm por toda la extensión de la huella expuesta

Tras esto, en ambos grupos se colocaran los implantes para reparación del tendón al lecho óseo y se usarán las suturas para realizar la reparación tendinosa con una técnica de doble fila de implantes. Para terminar se hará una nueva medición de la superficie de la huella ósea que queda expuesta, si procede.

4.6. Duración.

24 meses

12 meses de reclutamiento + 12 meses de seguimiento de los sujetos incluidos

4.7. Criterios de finalización.

Se finalizará el estudio cuando se haya realizado la última visita (V6) del último paciente incluido y se le haya realizado la RM.

4.8. Contabilización del medicamento.

No procede

4.9. Códigos de aleatorización/ciego.

A cada centro participante se suministrarán sobres cerrados y numerados secuencialmente que incluyan en su interior el grupo asignado a cada paciente. En su interior se identificará el grupo al que pertenece el paciente (tratamiento o control) según lo obtenido en un generador de números aleatorios, que ofrecerá una distribución aleatoria dicotómica y equilibrada de números igual al 120% de los participantes estimados para el estudio. La asignación se realizará estratificada por centro pero todos los sobres cerrados se harán de forma centralizada en el Hospital Universitario Ramón y Cajal.

Estos sobres se abrirán en el momento en que el cirujano, durante la intervención, confirme que le sujeto cumple todos los criterios de inclusión y ninguno de los de exclusión. Una vez finalizada la cirugía el investigador local se comunicará con el investigador principal y le informará sobre la inclusión del paciente y el grupo asignado.

Dadas las características del estudio este será ciego para el paciente y para el evaluador de las RM. No estará cegado para el cirujano, responsable de las evaluaciones clínicas preoperatorias. Las evaluaciones clínicas postoperatorias serán realizadas por investigadores distintos al cirujano.

Para el análisis numérico de los resultados del estudio se cegarán los datos respecto a la naturaleza de los grupos (control o tratamiento) para que el responsable del análisis de resultados no conozca el grupo de tratamiento.

4.10. Datos fuente.

Los datos a recoger en el estudio se obtendrán de la historia clínica del paciente, del protocolo operatorio y del informe de alta. Asimismo, se obtendrá una copia de la resonancia magnética de control al año para su evaluación ciega por un radiólogo externo al centro.

De estas fuentes se recogerán los siguientes datos para analizar:

Código del paciente, Fecha de nacimiento, sexo, talla y peso.

Fecha de intervención, así como de las siete visitas

Niveles de dolor: respuestas en las cuatro preguntas del BPI en las siete visitas.

Valores en la escala de Constant-Murley y EQ-5D-3L en las visitas 0, 4, 5 y 6.

En la resonancia previa: grado de atrofia del músculo supraespinoso/ infraespinoso/ subescapular/ redondo menor según la clasificación de Goutalier.

En el momento de la intervención: tamaño de la huella no cubierta por tendón evaluado en milímetros cuadrados antes de la reparación, retracción máxima del tendón, morfología de la lesión (según la clasificación de Burkhart [(Davidson and Burkhart 2010]). tamaño de la huella no cubierta por tendón

evaluado en milímetros cuadrados después de la reparación. Aparición de complicaciones intraoperatorias tales como avulsión de implantes, fracturas del troquíter, fallo parcial de la sutura tendinosa.

En la resonancia realizada al año: grado de atrofia del músculo supraespinoso/ infraespinoso/ subescapular/ redondo menor según la clasificación de Goutalier. Tamaño de la huella no cubierta por tendón evaluado en milímetros cuadrados. Integridad del tendón del supraespinoso: definido como una de las siguientes alternativas: **0** Normal, **1** Tendinosis (alteración de señal, sin signos de rotura), **2** Rotura de espesor parcial (cara bursal/cara articular), **3** Rotura de total.

Durante el seguimiento: evaluación de complicaciones postoperatorias tales como infección, rigidez o fractura.

4.11. Definición de final de ensayo clínico.

No está planificado la realización de análisis estadísticos intermedios.

Se finalizará el ensayo clínico tras la realización de la última visita y RM del último paciente incluido.

5. *SELECCIÓN Y RETIRADA DE SUJETOS*

5.1. Criterios de inclusión

- Pacientes, que tras haber recibir información sobre el diseño, los fines del estudio, los posibles riesgos que de él pueden derivarse y de que en cualquier momento pueden denegar su colaboración, otorguen por escrito su consentimiento para participar en el estudio.
- Ser mayor de 18 años.
- Pacientes que presenten:
- Roturas sintomáticas aisladas del tendón del músculo supraespinoso (SE)
- Con menos de 3cm de retracción,

- Con ausencia de atrofia muscular grave confirmada por resonancia magnética o TC (Ausencia de atrofia o atrofia grado I, II o III de Goutalier en la masa muscular)
- Que se confirme durante la artroscopia exploradora al iniciar la intervención.
- La RM o el TC deben de haberse realizado como máximo 4 meses antes de la intervención.
- No presencia de rotura que precise reparación en los tendones de los músculos subescapular ni infraespinoso
- Entender el propósito del estudio y estar disponibles para realizar frecuentes visitas al hospital.
- Prueba de embarazo en orina negativa realizada en los 7 días anteriores al comienzo del tratamiento en estudio en mujeres premenopáusicas o < 2 años después de la menopausia

5.2. Criterios de exclusión.

- Embarazo o planificación de quedarse embarazada durante el transcurso del estudio.
- Roturas del tendón SE de más de 3cm de retracción
- Roturas del tendón del SE que se consideren irreparables en le momento de la intervención
- Presencia de roturas de los tendones subescapular y/o infraespinoso
- Presencia de atrofia grado IV de Goutalier en masa muscular del supraespinoso
- Antecedentes de otras patologías ortopédicas en el hombro afecto (cirugías previas en el hombro, fracturas, re-roturas tendinosas, enfermedades reumáticas, artritis sépticas, …)

5.3. Número de sujetos previsto y justificación.

Por tratarse de un estudio piloto, se ha estimado el número mínimo de pacientes que es suficiente para probar el concepto que se pretende. Se ha realizado una predeterminación del tamaño muestral que se consigna en el apartado correspondiente.

5.4. Criterios de retirada y análisis.

El paciente podrá suspender su participación en el estudio en cualquier momento que lo desee.

A su juicio y criterio, el médico investigador podrá también decidir la retirada de un paciente del ensayo si éste no cumple las normas del protocolo.

El análisis de los datos se hará por intención de tratar.

5.5. Duración aproximada del periodo de reclutamiento.

12 meses.

6. *DESCRIPCIÓN DEL TRATAMIENTO*

6.1. Definición del tratamiento.

El producto se utilizará durante la intervención quirúrgica y tras la preparación de la huella ósea de inserción del tendón roto según las instrucciones del fabricante, realizando múltiples orificios de 1mm de diámetro en el hueso, separados 2-3 mm por toda la extensión de la huella expuesta,

El **NanoFx Microfracture Instrument** se introduce en el espacio subacromial del sujeto a través de uno de los portales artroscópicos tras hacer la preparación estándar de la huella de inserción del supraespinoso (resección de los restos tendinosos, exposición del hueso y regularización de éste). La pieza de mano se coloca sobre el lecho óseo y se impacta la aguja-broca en le interior del hueso. Se retira con ayuda del accesorio para extracción, se recoloca y se realizan las perforaciones necesarias según la extensión de la huella.

6.2. Tratamientos concomitantes.

Permitidos: cualquiera que requiera el paciente según el protocolo local

Prohibidos: ninguno

6.3. Cumplimiento.

El cirujano consignará en el parte quirúrgico de la intervención el resultado de la aleatorización asi como el adecuado uso del producto si correspondiese.

7. *EVALUACIÓN DE LA EFICACIA*

7.1. Criterios de valoración de eficacia.

<u>El producto se considerará eficaz si ocurre cualquiera de las siguientes:</u>

- Aparece una diferencia mayor del 10% respecto a la tasa de rotura (parcial o total) del tendón del supraespinoso en resonancia magnética al año de la reparación.
- Hay diferencias significativas y mayores del 10% del tamaño de la rotura en resonancia magnética evaluando el tamaño total de la huella de inserción del supraespinoso y la cantidad de esta (definida en porcentaje sobre la huella total) que queda descubierta (sin tejido tendinoso superpuesto) al año de la reparación.
- Hay diferencias significativas y mayores del 10% en los niveles de dolor medidos con las preguntas las preguntas 3 a 6 del Brief Pain Inventory.
- Hay diferencias significativas y mayores del 10 puntos en el resultado funcional medido con la Escala de Constant-Murley.

<u>El producto se considerará seguro si:</u>

- No aparecen diferencias significativas en la tasa de complicaciones identificadas en el estudio, se consideren relacionadas o no con el producto a estudio

7.2. Desarrollo del estudio.

A los pacientes en los que inicialmente se considere que son candidatos a cumplir los criterios de inclusión se les realizará los siguientes procedimientos:

- Historia clínica.
- Exploración física completa que incluirá talla y peso corporal.
- Evaluación de los niveles de dolor: mediante las preguntas 3 a 6 del Brief Pain Inventory.
- Evaluación funcional del hombro mediante la escala de Constant Murley
- Evaluación de la calidad de vida mediante la escala EQ-5D-3L

Tras su inclusión durante la cirugía, los pacientes serán distribuidos en dos grupos: tratamiento y control.

A continuación se realizará la sutura del tendón.

Los sujetos serán seguidos durante al menos 12 meses tras la cirugía. Durante este período se realizarán las evaluaciones indicadas arriba según este esquema.

ESQUEMA DEL ESTUDIO

Se estructura en 8 visitas:

Visita 0: Evaluación del paciente para su posible inclusión en el estudio. Programación de la IQ. Información y firma del CI.

Día intervención quirúrgica (IQ): evaluación intraoperatoria del cumplimiento de los criterios de inclusión. Confirmación de la ausencia de criterios de exclusión. Aleatorización intraoperatoria. Asignación a grupo de tratamiento o control. Intervención y uso o no del producto.

Visita 1: 5-10 días tras la cirugía

Visita 2: 18-24 días tras la cirugía

Visita 3: 6-8 semanas tras la cirugía

Visita 4: 3 meses (+/- 1 semana) tras la cirugía

Visita 5: + 6 meses(+/- 1 semana) tras la cirugía

Visita 6: +12 meses (+/- 1 semana) tras la cirugía. Realización de la RM control

Día	*V0*	*IQ*	*V1*	*V2*	*V3*	*V4*	*V5*	*V6*
**Consentimiento informado**	X							
**Historia clínica**	X				X	X	X	X
**Exploración**	X				X	X	X	X
**Criterios de inclusión**	X	X						
**Aleatorización**		X						
**Evaluación de dolor**	X		X	X	X	X	X	X
**Recogida de efectos adversos**		X	X	X	X	X	X	X
**Evaluación de complicaciones**		X	X	X	X	X	X	X
**Constant Murley**	X					X	X	X
**EQ-5D-3L**	X					X	X	X
**RM**	X							X

7.3. Descripción de los métodos para la valoración de la eficacia.

Se indican en le apartado 7.1

7.3.1. **Determinaciones de laboratorio.**

No se contempla la realización de ningún test analítico suplementario a los sujetos antes o después de la intervención aparte de los establecidos en el protocolo de evaluación preanestesica del centro

8. SEGURIDAD

8.1. DEFINICIONES

El documento "Guidelines on medical devices: Clinical investigations: Serious Adverse event reporting under Directives 90/385/EECC and 93/42/EEC", relativo a la seguridad en investigación clínica con productos sanitarios, define, según la relación y/o gravedad de los acontecimientos adversos, la siguiente clasificación:

- Acontecimiento adverso (AA)
- Efecto adverso del dispositivo (EAD)
- Acontecimiento adverso grave (AAG)
- Efecto adverso grave del dispositivo (EAGD)
- Efecto adverso grave imprevisto del dispositivo (EAGID)

8.1.1. Acontecimiento Adverso, AA (AE, adverse event)

Cualquier episodio médico no deseado, enfermedad o lesión no prevista, o signos clínicos no deseados (incluyendo los hallazgos de laboratorio anormales) en sujetos participantes en un ensayo clínico, estén o no relacionados con el producto sanitario en investigación o con el control.

8.1.2. Efecto Adverso del Producto, EAP (ADE, adverse device effect)

Acontecimiento adverso relacionado con la utilización de un producto sanitario en investigación.

Esta definición incluye Acontecimientos Adversos que resulten de la insuficiencia o inadecuación de las instrucciones de uso, despliegue, implantación, instalación o utilización, o de cualquier mal funcionamiento del producto sanitario en investigación o que resulte de un error de utilización o de la utilización indebida deliberada del producto sanitario en investigación.

8.1.3. Acontecimiento Adverso Grave, AAG (SAE, serious adverse event)

Acontecimiento adverso que:

a) Provoque la muerte;

b) Provoque un deterioro grave de la salud del sujeto que:

1) de lugar a una enfermedad o lesión con riesgo de pérdida de la vida, o
2) de lugar a una deficiencia permanente de una estructura corporal o una función corporal, o
3) la prolongación de la hospitalización existente, o
4) requiera una intervención médica o quirúrgica para impedir una enfermedad con riesgo de pérdida de la vida o lesión o deficiencia permanente de una estructura corporal o una función corporal,

c) Provoque sufrimiento fetal, muerte fetal o una anomalía congénita o un defecto del nacimiento.

La hospitalización planificada para una condición preexistente, o un procedimiento requerido por el protocolo, sin deterioro grave del estado de salud, no se considera un Acontecimiento Adverso Grave.

8.1.4. Efecto Adverso Grave del Producto en investigación, EAGP (SADE, serious adverse device effect)

Efecto adverso del producto en investigación que ha producido cualquier consecuencia característica de un Acontecimiento Adverso Grave.

8.1.5. Efecto adverso grave inesperado del producto, EAGIP (USADE, unanticipated Serious Adverse Device Effect)

Efecto Adverso Grave del producto de investigación que por su naturaleza, incidencia, intensidad o consecuencias no ha sido identificado en la versión actualizada del informe de análisis de riesgo (en este caso manual de instrucciones de uso).

Un Efecto Adverso Grave Esperado del Producto (EAGEP) es un efecto que por su naturaleza, intensidad o consecuencias ha sido identificado en la versión actualizada del informe de análisis de riesgo (en este caso manual de instrucciones de uso).

8.1.6. Deficiencia del producto

Inadecuación de un producto sanitario con respecto a su identidad, calidad, durabilidad, fiabilidad, seguridad o prestaciones. Las deficiencias del producto incluyen los fallos de funcionamiento, los errores de utilización, y la inadecuación del etiquetado.

8.2. Informacion de Seguridad de Referencia

En este estudio, la información de seguridad de referencia (ISR) será la información contenida en el Manual de instrucciones de uso del producto sanitario en investigación.

8.3. Intensidad

El término "intenso" se utiliza para describir la intensidad de un efecto. Un acontecimiento intenso puede tener una importancia médica relativamente menor, como una cefalea muy intensa. Esto no es lo mismo que cuando hablamos de un AA grave. El término "grave" sirve como guía para definir las obligaciones regulatorias de comunicación.

La intensidad de los AA se asignará siguiendo las definiciones siguientes:

Leve: El paciente es consciente de los síntomas pero son tolerados fácilmente o no precisan ningún tratamiento específico.

Moderado: El paciente presenta una molestia que interfiere con su actividad habitual y/o la condición precisa tratamiento específico.

Intenso: El paciente está incapacitado para el trabajo o para realizar sus actividades habituales y/o el acontecimiento requiere medidas terapéuticas significativas.

8.4. Causalidad

La relación del Acontecimiento adverso con el producto sanitario en investigación debe clasificarse como:

AA Relacionado: La relación temporal del AA con el producto sanitario en investigación indica una relación causal posible y no puede ser explicado por factores tales como el estado clínico del paciente, intervenciones terapéuticas.

Siguiendo las definiciones del apartado 8.1 corresponderían a los EAP y los EAGP.

AA no Relacionado: La relación temporal del AA con el producto sanitario en investigación indica una relación causal improbable, o bien otros factores (medicación o condiciones concomitantes), otras intervenciones terapéuticas proporcionan una explicación satisfactoria para el AA.

8.5. Procedimiento de recogida de los Acontecimientos Adversos

Los investigadores serán responsables de documentar todos los Acontecimientos Adversos en la historia clínica del paciente en base a lo referido por el paciente de manera espontánea o bien en respuesta al interrogatorio abierto del investigador en cada una de las visitas previstas en el calendario del estudio. Igualmente se evaluará y se dejará constancia en la historia clínica la relación de causalidad con la utilización del producto sanitario en investigación.

No obstante, está previsto recoger en el CRD los AA que sean valorados como relacionados con el uso del producto sanitario en investigación (EAP), así como aquellos AA que cumplan criterios de gravedad (EAGP) según se define en el apartado 8.1. También se recogerán todos los casos de muerte que ocurran durante el ensayo, independientemente de su relación con el Producto sanitario en investigación.

En el CRD se recogerá la siguiente información: descripción del acontecimiento, fecha de inicio y de fin del acontecimiento, intensidad, si está relacionado o no con el uso del producto sanitario en estudio tomada ante el acontecimiento, evolución o resultado del AA, y si es grave o no.

En caso de aparición de un acontecimiento adverso antes de la finalización de la participación en el ensayo, independientemente de su relación con el producto sanitario como de la gravedad del mismo, se realizará un seguimiento del paciente por parte del facultativo según la práctica asistencial habitual. Si se trata de un AA relacionado, grave o no grave, se anotarán en el CRD los datos de este seguimiento hasta la resolución o estabilización del mismo.

La relación entre los AAs y el producto sanitario en estudio será evaluada por parte del IP.

8.6. Notificacion de Acontecimientos Adversos

Dado que se trata de un estudio con un Producto Sanitario que posee marcado CE y que se utilizará bajo la indicación establecida en la evaluación de la conformidad, la vía de notificación de AA seguirá lo estipulado en el sistema de vigilancia descrito en el RD 1591/2009 y que se detalla en las Directrices de la AEMPS para la aplicación de dicho sistema por los centros y profesionales sanitarios (AEMPS/CTI-PS/octubre 2010).

El investigador principal de cada centro o quien delegue notificará al punto de vigilancia de productos sanitarios de la Comunidad Autónoma implicada y al promotor del estudio, aquellos AAG que cumplan con la definición de Incidente Adverso, es decir, cualquier funcionamiento defectuoso, fallo o deterioro de las características o del funcionamiento de un producto, así como cualquier deficiencia en el etiquetado o en las instrucciones de utilización que pueda o haya podido dar lugar a la muerte o al deterioro grave del estado de salud de un paciente o usuario.

La comunicación se hará tan pronto como sea posible cumplimentando el FORMULARIO DE NOTIFICACIÓN DE INCIDENTES POR LOS PROFESIONALES SANITARIOS que se encuentra en el anexo III de las directrices para la aplicación del sistema de vigilancia por los centros y profesionales sanitarios. La comunicación se podrá efectuar por correo electrónico, fax o correo postal, así como por vía telemática a través de las páginas web cuando esté disponible dicha vía. En el caso de incidentes muy graves también se podrá notificar por teléfono, enviando posteriormente el formulario por cualquiera de los medios indicados.

Los tipos de incidentes adversos que deben comunicarse son los siguientes:

1. Los que dan lugar a la muerte

2. Los que dan lugar a un deterioro grave del estado de salud del paciente, usuario u otra persona, como por ejemplo:
   a. Enfermedad o lesión con amenaza para la vida
   b. Deterioro permanente de una función corporal o daño permanente de una estructura corporal.
   c. Proceso que necesita una intervención médica o quirúrgica para evitar un deterioro permanente de una función corporal o un daño permanente de una estructura corporal.
3. Los incidentes potenciales, que son aquellos que podrían haber dado lugar a la muerte o deterioro graves de la salud, pero que no se han producido debido bien a circunstancias afortunadas o a la intervención de un profesional sanitario.

Desde las Comunidades Autónomas se remitirán a la Agencia Española de Medicamentos y Productos Sanitarios (AEMPS) los incidentes adversos recibidos.

8.7. Notificación Expeditiva de otra información de seguridad relevante

El promotor o quien delegue notificará tan pronto como sea posible después de que tenga conocimiento, cualquier información que pudiera modificar la relación beneficio/riesgo del uso del producto sanitario en investigación.

8.8. Embarazos

En el caso que ocurra un embarazo éste deberá seguirse para determinar el resultado, incluyendo finalización espontánea o voluntaria, detalles del nacimiento, y la presencia o ausencia de cualquier defecto de nacimiento, anomalía congénita, o complicaciones para la madre y/o el recién nacido. Cualquier incidente adverso que aparezca durante el embarazo debe ser registrado en el formulario de notificación de incidentes por los profesionales sanitarios.

9. ESTADÍSTICA

9.1. Cálculo del tamaño muestral.

Se realizó una predeterminación del tamaño maestral para estimar este número. Se uso la tasa de integridad evaluada en resonancia magnética como variable principal para este propósito. Estimando una tasa de integridad del 70% y para identificar una diferencia potencial entre los grupos de al menos el 20%, con un error alfa igual a 0.05 y una potencia (1-β) igual a 0.8 se consideraron necesarios 48 sujetos por brazo del estudio. Teniendo en cuenta una posible pérdida de sujetos del 15% durante el seguimiento se estableció un total de 60 sujetos.

9.2. Métodos estadísticos.

Al finalizar el ensayo se realizará un análisis de la normalidad de las variables cuantitativas.

Se realizarán los siguientes análisis:

1.-Se hará un análisis estadístico de los parámetros epidemiológicos, clínicos y de morfología de la lesión del supraespinoso (apreciada en la cirugía y en la RM preoperatoria) para definir la homogeneidad de los dos grupos

2.-Se harán comparaciones dos a dos de todos los parámetros clínicos y de RM postoperatorios.

3.-Se realizará asimismo un análisis multivariante usando todas las variables preoperatorios y el grupo de tratamiento como variables independientes y las dos variables primarias de valoración de resultado como variables dependientes.

El nivel de significación se establecerá en $p<0.05$

9.3. Criterios para la finalización del ensayo.

1.-La finalización del seguimiento clínico y radiológico de todos los pacientes incluidos

2.-La demora en el reclutamiento: se requiere un reclutamiento de al menos un 35% de los sujetos a los 6 meses del estudio, del 70% a los 12 meses o del 100% a los 18 meses para no declarar finalizado el estudio por falta de reclutamiento.

9.4. Pérdida de datos.

El monitor del estudio se encargará de contabilizar los datos perdidos, no utilizados y erróneos. A efectos del análisis estadístico los sujetos perdidos se contabilizarán según la mejor práctica.

9.5. Desviaciones del plan estadístico.

Todas las desviaciones del plan estadístico original será descrito y justificado ante el monitor del estudio y será anotado en el protocolo y en el informe final.

9.6. Selección de sujetos para el análisis.

Para el análisis 1 (ver punto 9.2) se incluirán todos los sujetos aleatorizados,

Para el análisis 2 y 3 se incluirán todos los pacientes con seguimiento mayor de un año no excluidos y que no hayan salido del estudio.

10. ACCESO A LOS DATOS/DOCUMENTOS FUENTE

Todos los datos recogidos para la realización del estudio, tanto para la elaboración de su *Historia clínica*, como para el resto de documentos del estudio, quedarán archivados en los Servicios participantes, en soporte papel y en formato informático. Las Historias clínicas se archivarán según normativa o procedimientos establecidos en el centro.

Los datos recogidos para el estudio estarán identificados mediante un código y solo el investigador principal/colaboradores podrán relacionar dichos datos con el paciente y con su historia clínica.

Los datos serán incluidos en una base de datos que sigue la Ley 15/1999 de Protección de Datos de Carácter Personal. Así mismo, la transmisión de datos se hará con las medidas de seguridad adecuadas en cumplimiento de dicha ley y el R.D. 1270/2007. Sólo aquellos datos de la historia clínica que estén relacionados con el estudio serán objeto de comprobación. Esta comprobación se hará en la medida de lo posible en presencia del Investigador Principal/Investigadores Colaboradores, responsables de garantizar la confidencialidad de todos los datos de las historias clínicas pertenecientes a los sujetos participantes en el ensayo clínico.

El acceso a esta información quedará restringido al promotor, médico del estudio/colaboradores, autoridades sanitarias, al Comité Ético de Investigación Clínica y personal autorizado por el promotor, cuando lo precisen para comprobar los datos y procedimientos del estudio, pero siempre manteniendo la confidencialidad de los mismos de acuerdo a la legislación vigente.

Tampoco se revelará la identidad del sujeto si se publican los resultados del estudio.

11. CONTROL Y GARANTÍA DE CALIDAD

Visitas de monitorización al centro:

Durante el estudio se realizarán al menos 2 visitas de monitorización para asegurar que se siguen todos los aspectos del protocolo. Se revisarán los documentos fuente para verificar la información registrada en los CRDs. Los documentos fuentes son, por definición, los documentos, datos y registros originales. El investigador y el centro garantizarán el acceso del promotor o su representante y del CEIC a los documentos originales.

Documentación y aspectos del estudio que pueden ser revisados: archivo del investigador, medicación del estudio, la historia clínica de los sujetos, la documentación del consentimiento informado, los CRDs y los documentos fuentes asociados. Es importante que el investigador y el personal del estudio se encuentren disponibles durante las visitas de supervisión y que se dedique tiempo suficiente al proceso.

Auditorias por Autoridades Sanitarias

Este estudio podrá ser inspeccionado por las autoridades sanitarias.

Si una autoridad sanitaria se pone en contacto con el centro de estudio para una inspección, deberá informarse inmediatamente al promotor. El investigador y el centro, deberán garantizar a los auditores el acceso a todos los documentos del estudio para la garantía de calidad.

12. *ASPECTOS ÉTICOS*

12.1. Normas generales y particulares para los investigadores

Los investigadores se atendrán estrictamente a lo dispuesto en este protocolo, cumplimentando totalmente las hojas de recogida de datos, que se enviarán a su debido tiempo al promotor o a la entidad colaboradora que éste designe para analizar los datos.

El ensayo se llevará a cabo de acuerdo a las recomendaciones para ensayos clínicos y evaluación de fármacos en el hombre, que figuran en la Declaración de Helsinki Brasil 2013 y en la actual Legislación Española en materia de ensayos clínicos.

12.2. Consentimiento informado

Todos los sujetos participantes antes de iniciar el estudio serán informados y darán su consentimiento por escrito. La hoja de consentimiento informado se incluye como documento anexo a este protocolo. Al voluntario se le entregará una copia de esta hoja de información para que se la lleve consigo.

12.3. Dispositivos de seguridad y confidencialidad

La información difundida y obtenida por la puesta en marcha del presente estudio es considerada confidencial y deberá ser tratada en todo momento como tal. Los sujetos del estudio se identificarán sólo con su código de sujeto en el estudio. Tanto los investigadores responsables del ensayo clínico, como un representante del promotor o de las Autoridades Sanitarias tendrán acceso a la información registrada a lo largo del estudio. En caso de publicación de los resultados del estudio no se revelará la identidad de los voluntarios.

12.4. Contenido del presupuesto del ensayo

No se prevé compensación económica a los investigadores o a los pacientes incluidos en el ensayo ya que no hay financiación para el estudio.

Asimismo, no se prevé que se incurra en ningún gasto suplementario en el manejo de los pacientes aparte del uso del producto, que será donado por el fabricante. Asimismo, las visitas programadas dentro del estudio comprenden las visitas que se consideran práctica habitual en los centros en los que este estudio se realiza. Por tanto, no se presenta memoria económica del proyecto.

13. *CONSIDERACIONES PRÁCTICAS*

13.1. Responsabilidades de los participantes del ensayo clínico

Los investigadores se atendrán a las normas de Buena Práctica Clínica y conocerán y seguirán los procedimientos normalizados de trabajo del Servicio .

Toda la información recogida durante la realización del ensayo se anotará directamente en el cuaderno de recogida de datos. Cuando se haga una corrección se deberá anotar la fecha y las iniciales de la persona que la realiza.

El personal auxiliar seguirá las instrucciones dadas por el investigador en cuanto a las extracciones de muestras de sangre, su manejo y demás exploraciones complementarias.

Previo al estudio, los pacientes deberán recibir información oral y escrita con respecto al diseño, fines del estudio y posibles riesgos que de él puedan derivarse . Si posteriormente acceden a participar en el mismo, deberán firmar su consentimiento, sin que ello impida que en cualquier momento y por cualquier razón puedan revocarlo y abandonar el estudio.

Los pacientes recibirán instrucciones respecto a la necesidad de respetar estrictamente las instrucciones de los investigadores. Se informará de la necesidad de ponerse en contacto con los investigadores si durante el estudio surgiera alguna incidencia, facilitándoles la forma de hacerlo durante el periodo ambulatorio del estudio.

13.2. Desviaciones del protocolo

Los investigadores presentes en tales circunstancias documentarán de forma completa la desviación y la razón en el CRD. En el caso de que la desviación tenga que ver con los criterios de inclusión/exclusión, los investigadores contactarán con el monitor clínico por teléfono a fin de informarle de tal desviación.

13.3. Archivo de la documentación

Existirá un archivo de documentación para todos los datos, que se conservarán íntegros en papel y en soporte informático durante al menos 25 años tras la finalización del estudio. Este archivo deberá contener los siguientes elementos:

1. Aprobación por el CEIC del protocolo y de la hoja de consentimiento informado.
2. Copia del impreso de consentimiento por escrito, y del protocolo aprobados con cualquier enmienda si procede.
3. Cualquier correspondencia relativa al estudio con el promotor, durante el transcurso del mismo.
4. Cualquier correspondencia con el CEIC.
5. Lista de miembros del CEIC que aprobó el protocolo del estudio.
6. Aceptación firmada del protocolo.
7. *Curriculum vitae* del investigador principal y de los otros investigadores que formen el equipo investigador.
8. Registro de firmas de los miembros del equipo investigador.
9. Comunicaciones de AA graves.
10. Contrato entre el promotor y el equipo investigador.
11. Lista de identidad de los pacientes.
12. Copias de los CRD.

La documentación será archivada siguiendo los procedimientos normalizados de trabajo del servicio de traumatología y cirugía ortopédica .

13.4. Enmiendas al protocolo

Ni el investigador ni el monitor ni el promotor modificarán este protocolo sin obtener previamente el consentimiento de las otras partes. La modificación debe documentarse por escrito. Cualquier cambio en la actividad de investigación, excepto los necesarios para eliminar un riesgo aparente inmediato para el paciente debe ser revisado y aprobado por el CEIC antes de su implantación. El promotor debe enviar las enmiendas al protocolo a las autoridades sanitarias, y las modificaciones pueden precisar la revisión y aprobación del CEIC.

13.5. Aceptación del investigador

El compromiso de investigador está incluido en el apéndice D.

13.6. Condiciones de publicación

Los resultados derivados del presente estudio serán publicados tanto positivos como negativos de acuerdo a lo que establece la normativa vigente, se requerirá la aprobación de los investigadores y de la entidad patrocinadora. El monitor y el promotor deberán disponer de una copia del manuscrito que se desea publicar 15 días antes de que éste se envíe al editor. Asimismo, se respetará siempre la confidencialidad de la identidad de los pacientes.

Antes de la inclusión de ningún paciente en el estudio, el estudio se dará de alta en el registro clinicaltrials.gov base de datos de acceso público. Donde permanecerá disponible para su consulta tanto para el público general como para el resto de la comunidad científica.

14. APÉNDICES

A. Preguntas 3 a 6 del Brief Pain Inventory

B. Escala de Constant-Murley para valoración de hombro.

C. Escala EQ-5D-3L

D. Compromiso del investigador
