# Supplementary material for: Footprint preparation with nanofractures in a supraspinatus repair cuts in half the retear rate at 1-year follow-up. A randomized controlled trial
Source: Knee Surg Sports Traumatol Arthrosc. 2020 Jun 1;29(7):2249–56. doi: 10.1007/s00167-020-06073-7 (PMC8225541; doi:10.1007/s00167-020-06073-7)
Supplement: Supplementary file 2 — Supplementary material 2 (PDF 1364 kb) [file 167_2020_6073_MOESM2_ESM.pdf]

Cuaderno de Recogida de Datos

MICROMANGUITO01

EFECTO EN EL ÍNDICE DE CICATRIZACIÓN DE LA REALIZACIÓN DE NANOFRACTURAS EN LA HUELLA ÓSEA DE REPARACIÓN DE ROTURAS DEL TENDÓN DEL SUPRAESPINOSO DEL HOMBRO

MICROMANGUITO01	Recomendaciones y normas

RECOMENDACIONES PARA LOS INVESTIGADORES

1. Lea atentamente el protocolo del estudio.
2. Compruebe que sus pacientes cumplen todos los criterios de inclusión y ninguno de los de exclusión.

NORMAS DE CUMPLIMENTACIÓN DEL CRD

1. Escriba con bolígrafo de **TINTA NEGRA**.
2. Escriba con letra clara y trazo firme.
3. **Responda a todas las preguntas, ninguna casilla debe quedar en blanco. Si no dispone de información, escriba NA (No Aplicable).**
4. Complete todas las fechas, indicando día, mes y año (dd/mm/aaaa) según lo indicado en el CRD.
5. No olvide firmar al final de cada visita.

CORRECCIÓN DE ERRORES

1. Tache con una simple línea horizontal el dato erróneo y escriba el correcto al lado.
2. Cada una de estas correcciones debe ser validada por medio de iniciales, firma y fecha.
3. No utilice líquido corrector.

MICROMANGUITO01	Nº Paciente	Visita Basal (VO)

Fecha de visita Basal: ___ ___ ___ (día mes año)

Consentimiento informado firmado

Fecha de la firma: ___ ___ ___ (día mes año)

Datos demográficos

Fecha de nacimiento: ___ ___ ___ (día mes año)

Sexo: ☐1 Varón
☐2 Mujer

Etnia: ☐1 Blanco (mediterráneo, caucásico, nórdico)
☐2 Otro (especificar):

____

Constantes Vitales

Por favor marque esta casilla si la evaluación **NO** se ha realizado: ☐

Peso : ______ kg

Talla : ______ cm

MICROMANGUITO01	Nº Paciente	Visita Basal (VO)

Historia Clínica / Enfermedades Concomitantes

¿El paciente ha padecido alguna enfermedad relevante, cirugía, accidentes? ☐ No ☐ Sí, especificar:

¿El paciente ha sido diagnosticado/padece síntomas al principio del estudio? ☐ No ☐ Sí, especificar:

	**Diagnóstico / Enfermedad relevante**	**Fecha inicio** *(parcial si es necesario)* día mes año	**Continua** 0 = No 1 = Sí
1			
2			
3			
4			
5			
6			
7			
8			
9			
10			
11			
12			
13			
14			
15			
16			
17			
18			
19			
20			

MICROMANGUITO01	__|__ Nº Paciente	Visita Basal (VO)

Exploración Física

¿Se ha realizado la evaluación? ☐ Sí ☐ No

Códigos de sistema corporal[1] (1-15)	*Complete para todas las evaluaciones corporales que son anormales. Especifique una anormalidad por línea:*
___	______________________________
___	______________________________
___	______________________________
___	______________________________
___	______________________________
___	______________________________
___	______________________________
___	______________________________
___	______________________________
___	______________________________
___	______________________________
___	______________________________
___	______________________________
___	______________________________
___	______________________________
___	______________________________
___	______________________________
___	______________________________
___	______________________________
___	______________________________

[1]Códigos de sistema corporal: 1=Aspecto general; 2=Piel; 3=Cuello (incluido tiroides); 4=Ojos; 5=Oídos y nariz; 6=Mamas; 7=Pulmones; 8=Corazón; 9=Abdomen; 10=Espalda; 11=Genitales externos: 12=Nódulos linfáticos; 13=Extremidades; 14=Sistema nervioso; 15=Otros

MICROMANGUITO01	|__|__| Nº Paciente	Visita Basal (VO)

Exploración física del hombro afectado

Balance auricular

Marque con un círculo los grados de movilidad del hombro

Rotación externa activa:
110º 90º 60º 45º 30º 0º

Rotación externa pasiva:
110º 90º 60º 45º 30º 0º

Rotación interna activa:
D12 L3 L5 Glúteo

Rotación interna pasiva:
D12 L3 L5 Glúteo

Flexión activa:
180º 150º 120º 90º 60º 30º 0º

Flexión pasiva:
180º 150º 120º 90º 60º 30º 0º

Abducción activa:
160º 120º 90º 45º 20º 0º

Abducción pasiva:
160º 120º 90º 45º 20º 0º

Manibras de Exploración:

Maniobra de Jobe	☐ Positiva	☐ Negativa
Maniobra de Yokum	☐ Positiva	☐ Negativa
Maniobra de Lift-off Test	☐ Positiva	☐ Negativa
Dolor presión art. AC	☐ Positiva	☐ Negativa
Maniobra de O'Brien	☐ Positiva	☐ Negativa

MICROMANGUITO01	Nº Paciente	Visita Basal (VO)

Radiografía del hombro

¿Se ha realizado la prueba? ☐ Sí ☐ No

Hombro: ☐ Derecha ☐ Izquierda

Fecha de realización: |_|_|_|_|_|_|_|_| Durante los seis meses anteriores al inicio del estudio
día mes año

Lesiones descritas:

☐ Calcificaciones espacio subacromial
☐ Artropatía gleno-humeral
☐ Artropatía acromio-clavicular

RNM Resonancia Nuclear Magnética

¿Se ha realizado la prueba? ☐ Sí ☐ No

Fecha de realización: |_|_|_|_|_|_|_|_| Durante los seis meses anteriores al inicio del estudio
día mes año

Marque con una cruz:

Rotura del tendón SE de menos de 3 cm.	☐ Sí	☐ No
Rotura de los tendones subescapular y/o infraespinoso	☐ Sí	☐ No
Signos de degeneración grasa G. I-III de Goutalier	☐ Sí	☐ No
Indemidad del tendón PLB	☐ Sí	☐ No

Grado de infiltración grasa de los tendones:

Supraespinoso	0	1	2	3	4
Infraespinoso	0	1	2	3	4
Subescapular	0	1	2	3	4

Grado de atrofia de los tendones:

Supraespinoso	0	1	2	3	4
Infraespinoso	0	1	2	3	4
Subescapular	0	1	2	3	4

Medicación concomitante

¿El paciente está recibiendo o ha recibido algún tratamiento en las últimas dos semanas? ☐ Sí ☐ No

En caso afirmativo, cumplimente la hoja de **medicación concomitante**.

¿Ha recibido tratamiento intraarticular (excepto los últimos 6 meses)? ☐ Sí ☐ No

En caso afirmativo, especificar

- ☐ Corticoides
- ☐ Ácido hialurónico
- ☐ Ozonoterapia
- ☐ Otros ____

MICROMANGUITO01	Nº Paciente	Visita Basal (VO)

Cuestionario de Salud "Brief Pain Inventory" (Preguntas de 3 a 6)
**INSTRUCCIONES:**<br>Las preguntas que siguen se refieren a lo que usted piensa sobre su salud. Sus respuestas permitirán saber cómo se encuentra usted y hasta qué punto es capaz de hacer sus actividades habituales. Conteste cada pregunta tal y como se indica. Si no está seguro/a de cómo responder a una pregunta, por favor conteste lo que le parezca más cierto.<br>Fecha de realización: __ __ __ (día mes año)<br>**MARQUE UNA SOLA RESPUESTA**
**1. Clasifique su dolor haciendo un círculo alrededor del número que mejor describe la intensidad MÁXIMA de dolor sentido en las últimas 24 horas.**
0 Ningún dolor — 1 — 2 — 3 — 4 — 5 — 6 — 7 — 8 — 9 — 10 Máximo dolor
**2. Clasifique su dolor haciendo un círculo alrededor del número que mejor describe la intensidad MÍNIMA de dolor sentido en las últimas 24 horas.**
0 Ningún dolor — 1 — 2 — 3 — 4 — 5 — 6 — 7 — 8 — 9 — 10 Máximo dolor
**3. Clasifique su dolor haciendo un círculo alrededor del número que mejor describe la intensidad MEDIA de dolor sentido.**
0 Ningún dolor — 1 — 2 — 3 — 4 — 5 — 6 — 7 — 8 — 9 — 10 Máximo dolor
**4. Clasifique su dolor haciendo un círculo alrededor del número que mejor describe la intensidad ACTUAL.**
0 Ningún dolor — 1 — 2 — 3 — 4 — 5 — 6 — 7 — 8 — 9 — 10 Máximo dolor

Cuestionario de Salud EQ-5D-3L

Marque con una cruz la respuesta de cada apartado que mejor describa su estado de salud el día de HOY.

Movilidad

No tengo problemas para caminar ❑

Tengo algunos problemas para caminar ❑

Tengo que estar en la cama ❑

Cuidado Personal

No tengo problemas con el cuidado personal ❑

Tengo algunos problemas para lavarme o vestirme ❑

Soy incapaz de lavarme o vestirme ❑

Actividades Cotidianas *(ej, trabajar, estudiar, hacer las tareas domésticas, actividades familiares o actividades durante el tiempo libre)*

No tengo problemas para realizar mis actividades cotidianas ❑

Tengo algunos problemas para realizar mis actividades cotidianas ❑

Soy incapaz de realizar mis actividades cotidianas ❑

Dolor / Malestar

No tengo dolor ni malestar ❑

Tengo moderado dolor o malestar ❑

Tengo mucho dolor o malestar ❑

Ansiedad / Depresión

No estoy ansioso ni deprimido ❑

Estoy moderadamente ansioso o deprimido ❑

Estoy muy ansioso o deprimido ❑

MICROMANGUITO01	Nº Paciente	Visita Basal (VO)

El mejor estado de salud imaginable

Para ayudar a la gente a describir lo bueno o malo que es su estado de salud hemos dibujado una escala parecida a un termómetro en el cual se marca con un 100 el mejor estado de salud que pueda imaginarse y con un 0 el peor estado de salud que pueda imaginarse.

Nos gustaría que nos indicara en esta escala, en su opinión, lo bueno o malo que es su estado de salud en el día de HOY. Por favor, dibuje una línea desde el casillero donde dice "Su estado de salud hoy" hasta el punto del termómetro que en su opinión indique lo bueno o malo que es su estado de salud en el día de HOY.

Su estado de salud hoy

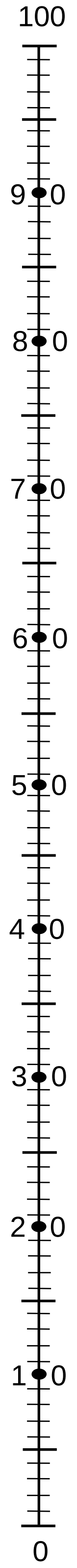

TEST DE CONSTANT-MURLEY:

A- DOLOR:

1- ¿Cuánto dolor tiene en el hombro en sus actividades de la vida diaria?	
0 puntos	Dolor severo o permanente
5 puntos	Dolor moderado
10 puntos	Dolor ligero
15 puntos	No dolor

2. Escala lineal:

Si "0" significa no tener dolor y "15" el mayor dolor que pueda sentir, haga un circulo sobre el nivel de dolor de su hombro.
La puntuación es inversamente proporcional a la la escala de dolor (Por ejemplo, un nivel de 5 son 10 puntos)

Nivel de dolor	0	1	2	3	4	5	6	7	8	9	10	11	12	13	14	15
Puntos	15	14	13	12	11	10	9	8	7	6	5	4	3	2	1	0

TOTAL A (1+2/2):

B- ACTIVIDADES DE LA VIDA DIARIA

1- ¿Está limitada la vida diaria por el hombro?

0 puntos	Limitación severa
2 puntos	Limitación moderada
4 puntos	Sin limitación

2- ¿Está limitada la vida deportiva por el hombro?

0 puntos	Limitación severa
2 puntos	Limitación moderada
4 puntos	Sin limitación

3- Sueño

0 puntos	El dolor impide dormir
1 punto	El dolor a veces despierta
2 puntos	Ninguna molestia

4- Altura a la que se puede elevar el brazo para coger un objeto

2 puntos	Altura de la cintura
4 puntos	Altura del xifoides
6 puntos	Altura del cuello
8 puntos	Altura de la cabeza
10 puntos	Encima de la cabeza

TOTAL B (1+2+3+4):

C- VALORACIÓN DEL PARÁMETRO MOVILIDAD	
**1- Flexión**	
0 puntos	0º - 30º
2 puntos	31º - 60º
4 puntos	61º - 90º
6 puntos	91º - 120º
8 puntos	121º - 150º
10 puntos	151º - 180º
**2- Abducción**	
0 puntos	0º - 30º
2 puntos	31º - 60º
4 puntos	61º - 90º
6 puntos	91º - 120º
8 puntos	121º - 150º
10 puntos	151º - 180º
**3- Rotación Externa**	
2 puntos	Mano detrás de la cabeza, codo adelante
4 puntos	Mano detrás de la cabeza, codo atrás
6 puntos	Mano sobre la cabeza, codo adelante
8 puntos	Mano sobre la cabeza, codo atrás
10 puntos	Elevación completa por encima de la cabeza
**4- Rotación Interna**	
0 puntos	Dorso de la mano en trocanter
2 puntos	Dorso de la mano en la nalga
4 puntos	Dorso de la mano en articulación sacro-ilíaca
6 puntos	Dorso de la mano en la cintura
8 puntos	Dorso de la mano en la vértebra dorsal 12
10 puntos	Dorso de la mano en la zona interescapular

TOTAL C (1+2+3+4):

D- VALORACIÓN DEL PARÁMETRO FUERZA

La fuerza se medirá con un dinamómetro (mecánico o electrónico), con el muelle fijado al suelo por un extremo, y la cincha fijada a la muñeca del paciente por el otro, con la extremidad superior en abducción a 90º en el plano escapular, el codo extendido y el antebrazo pronado.
El resultado vendrá dado por la mayor de tres mediciones consecutivas de 5 segundos de duración. Si el paciente no alcanza los 90º, la fuerza se mide en la máxima abducción que pueda conseguir.
Se puntúa hasta un máximo de 25 puntos (12,5kg)
La medición debe de ser libre de dolor, si el paciente tiene dolor al hacer fuerza puntúa 0 puntos.

TOTAL D (Media Kg. x 2):

TOTAL (A + B + C + D):

MICROMANGUITO01	Nº Paciente	Visita Basal (VO)

Criterios de inclusión

	Sí	No
1. Paciente mayor de 18 años	☐	☐
2. Rotura síntomática aislada del tendón supraespinoso (SE) con menos de 3 cm. de retracción, con ausencia de atrofia muscular grave	☐	☐
3. RM o TC realizados como máximo 6 meses antes de la intervención	☐	☐
4. No presencia de roturas que requieran la reparación de los tendones subescapular e infraespinoso	☐	☐
5. Consentimiento Informado por escrito del paciente.	☐	☐
6. El paciente es capaz de entender la naturaleza del estudio.	☐	☐
		⇑

Una casilla marcada en esta columna excluye al paciente del estudio

MICROMANGUITO01	|__|__| Nº Paciente	Visita Basal (VO)

Criterios de exclusión

		Sí	No
1.	Paciente < 18 años, o legalmente dependiente	☐	☐
2.	Paciente embarazada o planificación de quedarse embarazada durante el estudio, o en estado de lactancia	☐	☐
3.	Roturas del tendón SE que se consideren irreparables en el momento de la intervención	☐	☐
4.	Presencia de roturas de los tendones subescapular y/o infraespinoso	☐	☐
5.	Presencia de atrofia G.IV de Goutalier en la masa muscular del supraespinoso	☐	☐
6.	Antecedentes de otras patologías ortopédicas en el hombro afecto (cirugías previas en el hombro, fracturas, re-roturas tendinosas, enfermedades reumáticas, artritis sépticas, …)	☐	☐
7.	Enfermedad neoplásica	☐	☐
8.	Estados inmunodepresivos	☐	☐
9.	Participación simultánea en otro ensayo clínico o tratamiento con otro producto en fase de investigación en los 30 días previos a la inclusión en el estudio.	☐	☐
10.	Otras patologías o circunstancias que comprometan la participación en el estudio según criterio médico.	☐	☐
		⇑	

Una casilla marcada en esta columna excluye al paciente del estudio

Por la presente declaro que la información del paciente perteneciente al estudio cuyo código de identificación figura en estas páginas es fiel reflejo de la realidad.

____________________________________ \|__\|__\|__\|__\|__\|__\|__\|__\|

Firma investigador | Fecha Firma

MICROMANGUITO01	Nº Paciente	Intervención Quirúrgica (IQ)

Se realiza una nueva reevaluación de los criterios de inclusión y exclusión en el estudio una vez realizada la artroscopia exploradora

Criterios de inclusión

	Sí	No
1. Paciente mayor de 18 años	☐	☐
2. Rotura síntomática aislada del tendón supraespinoso (SE) con menos de 3 cm. de retracción, con ausencia de atrofia muscular grave	☐	☐
3. RM o TC realizados como máximo 6 meses antes de la intervención	☐	☐
4. No presencia de roturas que requieran la reparación de los tendones subescapular e infraespinoso	☐	☐
5. Consentimiento Informado por escrito del paciente.	☐	☐
6. El paciente es capaz de entender la naturaleza del estudio.	☐	☐
		⇑

Una casilla marcada en esta columna excluye al paciente del estudio

Criterios de exclusión

	Sí	No
1. Paciente < 18 años, o legalmente dependiente	☐	☐
2. Paciente embarazada o planificación de quedarse embarazada durante el estudio, o en estado de lactancia	☐	☐
3. Roturas del tendón SE que se consideren irreparables en el momento de la intervención	☐	☐
4. Presencia de roturas de los tendones subescapular y/o infraespinoso	☐	☐
5. Presencia de atrofia G.IV de Goutalier en la masa muscular del supraespinoso	☐	☐
6. Antecedentes de otras patologías ortopédicas en el hombro afecto (cirugías previas en el hombro, fracturas, re-roturas tendinosas, enfermedades reumáticas, artritis sépticas, …)	☐	☐
7. Enfermedad neoplásica	☐	☐
8. Estados inmunodepresivos	☐	☐
9. Participación simultánea en otro ensayo clínico o tratamiento con otro producto en fase de investigación en los 30 días previos a la inclusión en el estudio.	☐	☐
10. Otras patologías o circunstancias que comprometan la participación en el estudio según criterio médico.	☐	☐
	⇑	

Una casilla marcada en esta columna excluye al paciente del estudio

¿Se ha incluido al paciente en el estudio?	☐ Sí	☐ No
¿Se ha aleatorizado al paciente en el estudio?	☐ Sí	☐ No
Grupo de aleatorización	☐ NanoFx	☐ Control

MICROMANGUITO01	Nº Paciente	Intervención Quirúrgica (IQ)

CARACTERÍSTICAS DEL PROCEDIMIENTO QUIRÚRGICO
Marque con una cruz:

Posición del paciente
- ☐ Decúbito lateral
- ☐ Silla de playa

Anestesia
- ☐ General con plexo interescalénico
- ☐ General sin plexo interescalénico
- ☐ Sedación con plexo interescalénico
- ☐ Otras técnicas

Técnica de sutura del tendón SE
- ☐ Doble hilera con implantes roscados
- ☐ Doble hilera con implantes "todo sutura"
- ☐ Doble hilera según técnica "TOE"

Procedimientos asociados
- ☐ Resección clavícula distal
- ☐ Acromioplastia
- ☐ Tenotomía del bíceps
- ☐ Tenodesis del bíceps

Complicaciones intra-operatorias
- ☐ No
- ☐ Si (definir)

Tipo de Rotura: Crescent en "L" En "U"

Tamaño medio-lateral (retracción) máximo de la rotura (desde le borde lateral de la huella) en mm:	
Tamaño anteroposterior máximo de la rotura en mm	

Descripción de la lesión (indicar número de implantes y técnica utilizada).

Dibujar sobre la silueta:
- El patrón de la rotura
- Huella expuesta al terminar de preparar la huella,
- Localización de los implantes
- Huella cubierta con tendón al finalizar la reparación.

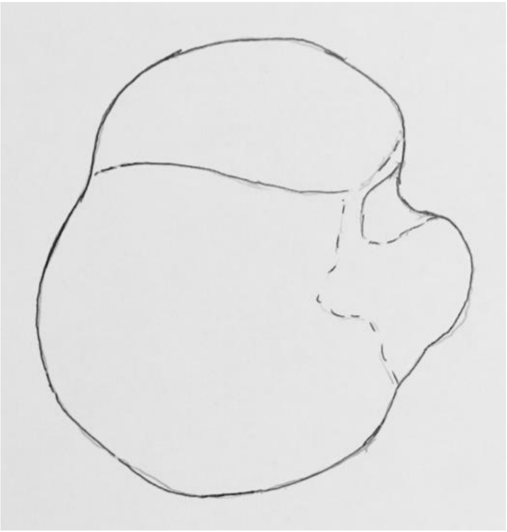

MICROMANGUITO01	|___|___| Nº Paciente	Intervención Quirúrgica (IQ)

Medicación concomitante

¿El paciente ha recibido algún tratamiento desde la última visita? ☐ Sí ☐ No

En caso afirmativo, cumplimente la hoja de **medicación concomitante**.

Acontecimientos Adversos

¿El paciente ha presentado algún Acontecimiento Adverso desde la última visita? ☐ Sí ☐ No

En caso afirmativo, cumplimente la hoja de **Acontecimientos Adversos**.

MICROMANGUITO01	Nº Paciente	Visita 1 (Día 5-10 post-IQ)

¿Ha acudido el paciente a la visita?	☐ Sí ☐ No
En caso **negativo**, ¿ha abandonado el paciente el estudio?	☐ Sí, cumplimentar hoja **Final del estudio**
	☐ No, especificar razón.<br>☐1 Acontecimientos Adversos<br>☐2 Razones personales<br>☐3 Otros, especificar: ______
Fecha de visita 1: día mes año	

Cuestionario de Salud "Brief Pain Inventory" (Preguntas de 3 a 6)

INSTRUCCIONES:

Las preguntas que siguen se refieren a lo que usted piensa sobre su salud. Sus respuestas permitirán saber cómo se encuentra usted y hasta qué punto es capaz de hacer sus actividades habituales. Conteste cada pregunta tal y como se indica. Si no está seguro/a de cómo responder a una pregunta, por favor conteste lo que le parezca más cierto.

Fecha de realización: día mes año

MARQUE UNA SOLA RESPUESTA

1. Clasifique su dolor haciendo un círculo alrededor del número que mejor describe la intensidad MÁXIMA de dolor sentido en las últimas 24 horas.

0	1	2	3	4	5	6	7	8	9	10
Ningún dolor										Máximo dolor

2. Clasifique su dolor haciendo un círculo alrededor del número que mejor describe la intensidad MÍNIMA de dolor sentido en las últimas 24 horas.

0	1	2	3	4	5	6	7	8	9	10
Ningún dolor										Máximo dolor

3. Clasifique su dolor haciendo un círculo alrededor del número que mejor describe la intensidad MEDIA de dolor sentido.

0	1	2	3	4	5	6	7	8	9	10
Ningún dolor										Máximo dolor

4. Clasifique su dolor haciendo un círculo alrededor del número que mejor describe la intensidad ACTUAL.

0	1	2	3	4	5	6	7	8	9	10
Ningún dolor										Máximo dolor

MICROMANGUITO01	|__|__| Nº Paciente	**Visita 1 (Día 5-10 post-IQ)**

Medicación concomitante

¿El paciente ha recibido algún tratamiento desde la última visita? ☐ Sí ☐ No

En caso afirmativo, cumplimente la hoja de **medicación concomitante**.

Acontecimientos Adversos

¿El paciente ha presentado algún Acontecimiento Adverso desde la última visita? ☐ Sí ☐ No

En caso afirmativo, cumplimente la hoja de **Acontecimientos Adversos**.

Evaluación de complicaciones

¿El paciente ha presentado alguna complicación desde la última visita? ☐ Sí ☐ No

En caso afirmativo, definir el tipo de complicación y su tratamiento.

**MICROMANGUITO01**	Nº Paciente	**Visita 2 (Día 18-24 post-IQ)**

¿Ha acudido el paciente a la visita? ☐ Sí ☐ No

En caso **negativo**, ¿ha abandonado el paciente el estudio? ☐ Sí, cumplimentar hoja **Final del estudio**

☐ No, especificar razón.
- ☐1 Acontecimientos Adversos
- ☐2 Razones personales
- ☐3 Otros, especificar: ______________________________

Fecha de visita 2: día mes año

**Cuestionario de Salud "Brief Pain Inventory" (Preguntas de 3 a 6)**
**INSTRUCCIONES:**<br>Las preguntas que siguen se refieren a lo que usted piensa sobre su salud. Sus respuestas permitirán saber cómo se encuentra usted y hasta qué punto es capaz de hacer sus actividades habituales. Conteste cada pregunta tal y como se indica. Si no está seguro/a de cómo responder a una pregunta, por favor conteste lo que le parezca más cierto.<br>Fecha de realización: día mes año<br>**MARQUE UNA SOLA RESPUESTA**
**1. Clasifique su dolor haciendo un círculo alrededor del número que mejor describe la intensidad MÁXIMA de dolor sentido en las últimas 24 horas.**
0 1 2 3 4 5 6 7 8 9 10<br>Ningún dolor … Máximo dolor
**2. Clasifique su dolor haciendo un círculo alrededor del número que mejor describe la intensidad MÍNIMA de dolor sentido en las últimas 24 horas.**
0 1 2 3 4 5 6 7 8 9 10<br>Ningún dolor … Máximo dolor
**3. Clasifique su dolor haciendo un círculo alrededor del número que mejor describe la intensidad MEDIA de dolor sentido.**
0 1 2 3 4 5 6 7 8 9 10<br>Ningún dolor … Máximo dolor
**4. Clasifique su dolor haciendo un círculo alrededor del número que mejor describe la intensidad ACTUAL.**
0 1 2 3 4 5 6 7 8 9 10<br>Ningún dolor … Máximo dolor

Medicación concomitante

¿El paciente ha recibido algún tratamiento desde la última visita? ☐ Sí ☐ No

En caso afirmativo, cumplimente la hoja de **medicación concomitante**.

Acontecimientos Adversos

¿El paciente ha presentado algún Acontecimiento Adverso desde la última visita? ☐ Sí ☐ No

En caso afirmativo, cumplimente la hoja de **Acontecimientos Adversos**.

Evaluación de complicaciones

¿El paciente ha presentado alguna complicación desde la última visita? ☐ Sí ☐ No

En caso afirmativo, definir el tipo de complicación y su tratamiento.

MICROMANGUITO01	__|__ Nº Paciente	**Visita 3 (6-8 semanas post-IQ)**

¿Ha acudido el paciente a la visita? ☐ Sí ☐ No

En caso **negativo**, ¿ha abandonado el paciente el estudio? ☐ Sí, cumplimentar hoja **Final del estudio**

☐ No, especificar razón.

- ☐1 Acontecimientos Adversos
- ☐2 Razones personales
- ☐3 Otros, especificar: ______________________________

Fecha de visita 3: __\|__\|____ (día mes año)

Cuestionario de Salud "Brief Pain Inventory" (Preguntas de 3 a 6)

INSTRUCCIONES:

Las preguntas que siguen se refieren a lo que usted piensa sobre su salud. Sus respuestas permitirán saber cómo se encuentra usted y hasta qué punto es capaz de hacer sus actividades habituales. Conteste cada pregunta tal y como se indica. Si no está seguro/a de cómo responder a una pregunta, por favor conteste lo que le parezca más cierto.

Fecha de realización: __\|__\|____ (día mes año)

MARQUE UNA SOLA RESPUESTA

1. Clasifique su dolor haciendo un círculo alrededor del número que mejor describe la intensidad MÁXIMA de dolor sentido en las últimas 24 horas.

0	1	2	3	4	5	6	7	8	9	10
Ningún dolor										Máximo dolor

2. Clasifique su dolor haciendo un círculo alrededor del número que mejor describe la intensidad MÍNIMA de dolor sentido en las últimas 24 horas.

0	1	2	3	4	5	6	7	8	9	10
Ningún dolor										Máximo dolor

3. Clasifique su dolor haciendo un círculo alrededor del número que mejor describe la intensidad MEDIA de dolor sentido.

0	1	2	3	4	5	6	7	8	9	10
Ningún dolor										Máximo dolor

4. Clasifique su dolor haciendo un círculo alrededor del número que mejor describe la intensidad ACTUAL.

0	1	2	3	4	5	6	7	8	9	10
Ningún dolor										Máximo dolor

MICROMANGUITO01	Nº Paciente	Visita 3 (6-8 semanas post-IQ)

Medicación concomitante

¿El paciente ha recibido algún tratamiento desde la última visita? ☐ Sí ☐ No

En caso afirmativo, cumplimente la hoja de **medicación concomitante**.

Acontecimientos Adversos

¿El paciente ha presentado algún Acontecimiento Adverso desde la última visita? ☐ Sí ☐ No

En caso afirmativo, cumplimente la hoja de **Acontecimientos Adversos**.

Evaluación de complicaciones

¿El paciente ha presentado alguna complicación desde la última visita? ☐ Sí ☐ No

En caso afirmativo, definir el tipo de complicación y su tratamiento.

MICROMANGUITO01	Nº Paciente	Visita 4 (3 meses +/- 1 semana post-IQ))

¿Ha acudido el paciente a la visita?	☐ Sí ☐ No
En caso **negativo**, ¿ha abandonado el paciente el estudio?	☐ Sí, cumplimentar hoja **Final del estudio**
	☐ No, especificar razón.<br>☐1 Acontecimientos Adversos<br>☐2 Razones personales<br>☐3 Otros, especificar:<br>_________________________
Fecha de visita 4: __/__/____ (día mes año)	

Cuestionario de Salud "Brief Pain Inventory" (Preguntas de 3 a 6)

INSTRUCCIONES:

Las preguntas que siguen se refieren a lo que usted piensa sobre su salud. Sus respuestas permitirán saber cómo se encuentra usted y hasta qué punto es capaz de hacer sus actividades habituales. Conteste cada pregunta tal y como se indica. Si no está seguro/a de cómo responder a una pregunta, por favor conteste lo que le parezca más cierto.

Fecha de realización: __/__/____ (día mes año)

MARQUE UNA SOLA RESPUESTA

1. Clasifique su dolor haciendo un círculo alrededor del número que mejor describe la intensidad MÁXIMA de dolor sentido en las últimas 24 horas.

0	1	2	3	4	5	6	7	8	9	10
Ningún dolor										Máximo dolor

2. Clasifique su dolor haciendo un círculo alrededor del número que mejor describe la intensidad MÍNIMA de dolor sentido en las últimas 24 horas.

0	1	2	3	4	5	6	7	8	9	10
Ningún dolor										Máximo dolor

3. Clasifique su dolor haciendo un círculo alrededor del número que mejor describe la intensidad MEDIA de dolor sentido.

0	1	2	3	4	5	6	7	8	9	10
Ningún dolor										Máximo dolor

4. Clasifique su dolor haciendo un círculo alrededor del número que mejor describe la intensidad ACTUAL.

0	1	2	3	4	5	6	7	8	9	10
Ningún dolor										Máximo dolor

Cuestionario de Salud EQ-5D-3L

Marque con una cruz la respuesta de cada apartado que mejor describa su estado de salud el día de HOY.

Movilidad

No tengo problemas para caminar ❑

Tengo algunos problemas para caminar ❑

Tengo que estar en la cama ❑

Cuidado Personal

No tengo problemas con el cuidado personal ❑

Tengo algunos problemas para lavarme o vestirme ❑

Soy incapaz de lavarme o vestirme ❑

Actividades Cotidianas *(ej, trabajar, estudiar, hacer las tareas domésticas, actividades familiares o actividades durante el tiempo libre)*

No tengo problemas para realizar mis actividades cotidianas ❑

Tengo algunos problemas para realizar mis actividades cotidianas ❑

Soy incapaz de realizar mis actividades cotidianas ❑

Dolor / Malestar

No tengo dolor ni malestar ❑

Tengo moderado dolor o malestar ❑

Tengo mucho dolor o malestar ❑

Ansiedad / Depresión

No estoy ansioso ni deprimido ❑

Estoy moderadamente ansioso o deprimido ❑

Estoy muy ansioso o deprimido ❑

MICROMANGUITO01	Nº Paciente	Visita 4 (3 meses +/- 1 semana post-IQ))

El mejor estado de salud imaginable

Para ayudar a la gente a describir lo bueno o malo que es su estado de salud hemos dibujado una escala parecida a un termómetro en el cual se marca con un 100 el mejor estado de salud que pueda imaginarse y con un 0 el peor estado de salud que pueda imaginarse.

Nos gustaría que nos indicara en esta escala, en su opinión, lo bueno o malo que es su estado de salud en el día de HOY. Por favor, dibuje una línea desde el casillero donde dice "Su estado de salud hoy" hasta el punto del termómetro que en su opinión indique lo bueno o malo que es su estado de salud en el día de HOY.

Su estado de salud hoy

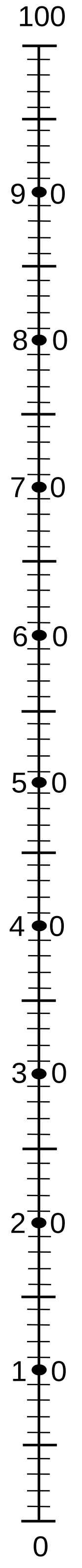

TEST DE CONSTANT-MURLEY:

A- DOLOR:

1- ¿Cuánto dolor tiene en el hombro en sus actividades de la vida diaria?	
0 puntos	Dolor severo o permanente
5 puntos	Dolor moderado
10 puntos	Dolor ligero
15 puntos	No dolor

2. Escala lineal:

Si "0" significa no tener dolor y "15" el mayor dolor que pueda sentir, haga un circulo sobre el nivel de dolor de su hombro.
La puntuación es inversamente proporcional a la la escala de dolor (Por ejemplo, un nivel de 5 son 10 puntos)

Nivel de dolor	0	1	2	3	4	5	6	7	8	9	10	11	12	13	14	15
Puntos	15	14	13	12	11	10	9	8	7	6	5	4	3	2	1	0

TOTAL A (1+2/2):

MICROMANGUITO01	|___|___| Nº Paciente	Visita 4 (3 meses +/- 1 semana post-IQ))

B- ACTIVIDADES DE LA VIDA DIARIA

1- ¿Está limitada la vida diaria por el hombro?

0 puntos	Limitación severa
2 puntos	Limitación moderada
4 puntos	Sin limitación

2- ¿Está limitada la vida deportiva por el hombro?

0 puntos	Limitación severa
2 puntos	Limitación moderada
4 puntos	Sin limitación

3- Sueño

0 puntos	El dolor impide dormir
1 punto	El dolor a veces despierta
2 puntos	Ninguna molestia

4- Altura a la que se puede elevar el brazo para coger un objeto

2 puntos	Altura de la cintura
4 puntos	Altura del xifoides
6 puntos	Altura del cuello
8 puntos	Altura de la cabeza
10 puntos	Encima de la cabeza

TOTAL B (1+2+3+4):

C- VALORACIÓN DEL PARÁMETRO MOVILIDAD	
**1- Flexión**	
0 puntos	0º - 30º
2 puntos	31º - 60º
4 puntos	61º - 90º
6 puntos	91º - 120º
8 puntos	121º - 150º
10 puntos	151º - 180º
**2- Abducción**	
0 puntos	0º - 30º
2 puntos	31º - 60º
4 puntos	61º - 90º
6 puntos	91º - 120º
8 puntos	121º - 150º
10 puntos	151º - 180º
**3- Rotación Externa**	
2 puntos	Mano detrás de la cabeza, codo adelante
4 puntos	Mano detrás de la cabeza, codo atrás
6 puntos	Mano sobre la cabeza, codo adelante
8 puntos	Mano sobre la cabeza, codo atrás
10 puntos	Elevación completa por encima de la cabeza
**4- Rotación Interna**	
0 puntos	Dorso de la mano en trocanter
2 puntos	Dorso de la mano en la nalga
4 puntos	Dorso de la mano en articulación sacro-ilíaca
6 puntos	Dorso de la mano en la cintura
8 puntos	Dorso de la mano en la vértebra dorsal 12
10 puntos	Dorso de la mano en la zona interescapular

TOTAL C (1+2+3+4):

D- VALORACIÓN DEL PARÁMETRO FUERZA

La fuerza se medirá con un dinamómetro (mecánico o electrónico), con el muelle fijado al suelo por un extremo, y la cincha fijada a la muñeca del paciente por el otro, con la extremidad superior en abducción a 90º en el plano escapular, el codo extendido y el antebrazo pronado.
El resultado vendrá dado por la mayor de tres mediciones consecutivas de 5 segundos de duración. Si el paciente no alcanza los 90º, la fuerza se mide en la máxima abducción que pueda conseguir.
Se puntúa hasta un máximo de 25 puntos (12,5kg)
La medición debe de ser libre de dolor, si el paciente tiene dolor al hacer fuerza puntúa 0 puntos.

TOTAL D (Media Kg. x 2):

TOTAL (A + B + C + D):

MICROMANGUITO01	|__|__| Nº Paciente	**Visita 4 (3 meses +/- 1 semana post-IQ))**

**Medicación concomitante**		
¿El paciente ha recibido algún tratamiento desde la última visita?	☐ Sí	☐ No
En caso afirmativo, cumplimente la hoja de **medicación concomitante**.		

**Acontecimientos Adversos**		
¿El paciente ha presentado algún Acontecimiento Adverso desde la última visita?	☐ Sí	☐ No
En caso afirmativo, cumplimente la hoja de **Acontecimientos Adversos**.		

**Evaluación de complicaciones**		
¿El paciente ha presentado alguna complicación desde la última visita?	☐ Sí	☐ No
En caso afirmativo, definir el tipo de complicación y su tratamiento.		

MICROMANGUITO01	__|__|__ Nº Paciente	**Visita 5 (+ 6 meses post-IQ)**

¿Ha acudido el paciente a la visita? ☐ Sí ☐ No

En caso **negativo**, ¿ha abandonado el paciente el estudio? ☐ Sí, cumplimentar hoja **Final del estudio**

☐ No, especificar razón.
- ☐1 Acontecimientos Adversos
- ☐2 Razones personales
- ☐3 Otros, especificar:

____

Fecha de visita 5: __|__|__|__|__|__|__|__ (día mes año)

Cuestionario de Salud "Brief Pain Inventory" (Preguntas de 3 a 6)

INSTRUCCIONES:
Las preguntas que siguen se refieren a lo que usted piensa sobre su salud. Sus respuestas permitirán saber cómo se encuentra usted y hasta qué punto es capaz de hacer sus actividades habituales. Conteste cada pregunta tal y como se indica. Si no está seguro/a de cómo responder a una pregunta, por favor conteste lo que le parezca más cierto.

Fecha de realización: __|__|__|__|__|__|__|__ (día mes año)

MARQUE UNA SOLA RESPUESTA

1. Clasifique su dolor haciendo un círculo alrededor del número que mejor describe la intensidad MÁXIMA de dolor sentido en las últimas 24 horas.

0 Ningún dolor	1	2	3	4	5	6	7	8	9	10 Máximo dolor

2. Clasifique su dolor haciendo un círculo alrededor del número que mejor describe la intensidad MÍNIMA de dolor sentido en las últimas 24 horas.

0 Ningún dolor	1	2	3	4	5	6	7	8	9	10 Máximo dolor

3. Clasifique su dolor haciendo un círculo alrededor del número que mejor describe la intensidad MEDIA de dolor sentido.

0 Ningún dolor	1	2	3	4	5	6	7	8	9	10 Máximo dolor

4. Clasifique su dolor haciendo un círculo alrededor del número que mejor describe la intensidad ACTUAL.

0 Ningún dolor	1	2	3	4	5	6	7	8	9	10 Máximo dolor

Cuestionario de Salud EQ-5D-3L

Marque con una cruz la respuesta de cada apartado que mejor describa su estado de salud el día de HOY.

Movilidad

No tengo problemas para caminar ❑

Tengo algunos problemas para caminar ❑

Tengo que estar en la cama ❑

Cuidado Personal

No tengo problemas con el cuidado personal ❑

Tengo algunos problemas para lavarme o vestirme ❑

Soy incapaz de lavarme o vestirme ❑

Actividades Cotidianas *(ej, trabajar, estudiar, hacer las tareas domésticas, actividades familiares o actividades durante el tiempo libre)*

No tengo problemas para realizar mis actividades cotidianas ❑

Tengo algunos problemas para realizar mis actividades cotidianas ❑

Soy incapaz de realizar mis actividades cotidianas ❑

Dolor / Malestar

No tengo dolor ni malestar ❑

Tengo moderado dolor o malestar ❑

Tengo mucho dolor o malestar ❑

Ansiedad / Depresión

No estoy ansioso ni deprimido ❑

Estoy moderadamente ansioso o deprimido ❑

Estoy muy ansioso o deprimido ❑

MICROMANGUITO01	Nº Paciente	**Visita 5 (+ 6 meses post-IQ)**

El mejor estado de salud imaginable

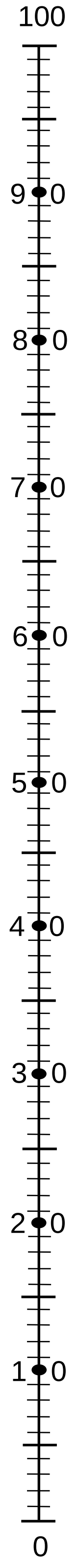

Para ayudar a la gente a describir lo bueno o malo que es su estado de salud hemos dibujado una escala parecida a un termómetro en el cual se marca con un 100 el mejor estado de salud que pueda imaginarse y con un 0 el peor estado de salud que pueda imaginarse.

Nos gustaría que nos indicara en esta escala, en su opinión, lo bueno o malo que es su estado de salud en el día de HOY. Por favor, dibuje una línea desde el casillero donde dice "Su estado de salud hoy" hasta el punto del termómetro que en su opinión indique lo bueno o malo que es su estado de salud en el día de HOY.

Su estado de salud hoy

MICROMANGUITO01	|__|__| Nº Paciente	Visita 5<br>(+ 6 meses post-IQ)

TEST DE CONSTANT-MURLEY:

A- DOLOR:

1- ¿Cuánto dolor tiene en el hombro en sus actividades de la vida diaria?	
0 puntos	Dolor severo o permanente
5 puntos	Dolor moderado
10 puntos	Dolor ligero
15 puntos	No dolor

2. Escala lineal:

Si "0" significa no tener dolor y "15" el mayor dolor que pueda sentir, haga un circulo sobre el nivel de dolor de su hombro.
La puntuación es inversamente proporcional a la la escala de dolor (Por ejemplo, un nivel de 5 son 10 puntos)

Nivel de dolor	0	1	2	3	4	5	6	7	8	9	10	11	12	13	14	15
Puntos	15	14	13	12	11	10	9	8	7	6	5	4	3	2	1	0

TOTAL A (1+2/2):

B- ACTIVIDADES DE LA VIDA DIARIA	
**1- ¿Está limitada la vida diaria por el hombro?**	
0 puntos	Limitación severa
2 puntos	Limitación moderada
4 puntos	Sin limitación
**2- ¿Está limitada la vida deportiva por el hombro?**	
0 puntos	Limitación severa
2 puntos	Limitación moderada
4 puntos	Sin limitación
**3- Sueño**	
0 puntos	El dolor impide dormir
1 punto	El dolor a veces despierta
2 puntos	Ninguna molestia
**4- Altura a la que se puede elevar el brazo para coger un objeto**	
2 puntos	Altura de la cintura
4 puntos	Altura del xifoides
6 puntos	Altura del cuello
8 puntos	Altura de la cabeza
10 puntos	Encima de la cabeza

TOTAL B (1+2+3+4):

MICROMANGUITO01	Nº Paciente	Visita 5 (+ 6 meses post-IQ)

C- VALORACIÓN DEL PARÁMETRO MOVILIDAD

1- Flexión

0 puntos	0º - 30º
2 puntos	31º - 60º
4 puntos	61º - 90º
6 puntos	91º - 120º
8 puntos	121º - 150º
10 puntos	151º - 180º

2- Abducción

0 puntos	0º - 30º
2 puntos	31º - 60º
4 puntos	61º - 90º
6 puntos	91º - 120º
8 puntos	121º - 150º
10 puntos	151º - 180º

3- Rotación Externa

2 puntos	Mano detrás de la cabeza, codo adelante
4 puntos	Mano detrás de la cabeza, codo atrás
6 puntos	Mano sobre la cabeza, codo adelante
8 puntos	Mano sobre la cabeza, codo atrás
10 puntos	Elevación completa por encima de la cabeza

4- Rotación Interna

0 puntos	Dorso de la mano en trocanter
2 puntos	Dorso de la mano en la nalga
4 puntos	Dorso de la mano en articulación sacro-ilíaca
6 puntos	Dorso de la mano en la cintura
8 puntos	Dorso de la mano en la vértebra dorsal 12
10 puntos	Dorso de la mano en la zona interescapular

TOTAL C (1+2+3+4):

D- VALORACIÓN DEL PARÁMETRO FUERZA

La fuerza se medirá con un dinamómetro (mecánico o electrónico), con el muelle fijado al suelo por un extremo, y la cincha fijada a la muñeca del paciente por el otro, con la extremidad superior en abducción a 90º en el plano escapular, el codo extendido y el antebrazo pronado.
El resultado vendrá dado por la mayor de tres mediciones consecutivas de 5 segundos de duración. Si el paciente no alcanza los 90º, la fuerza se mide en la máxima abducción que pueda conseguir.
Se puntúa hasta un máximo de 25 puntos (12,5kg)
La medición debe de ser libre de dolor, si el paciente tiene dolor al hacer fuerza puntúa 0 puntos.

TOTAL D (Media Kg. x 2):

TOTAL (A + B + C + D):

Medicación concomitante

¿El paciente ha recibido algún tratamiento desde la última visita? ☐ Sí ☐ No

En caso afirmativo, cumplimente la hoja de **medicación concomitante**.

Acontecimientos Adversos

¿El paciente ha presentado algún Acontecimiento Adverso desde la última visita? ☐ Sí ☐ No

En caso afirmativo, cumplimente la hoja de **Acontecimientos Adversos**.

Evaluación de complicaciones

¿El paciente ha presentado alguna complicación desde la última visita? ☐ Sí ☐ No

En caso afirmativo, definir el tipo de complicación y su tratamiento.

MICROMANGUITO01	Nº Paciente	Visita 6 (+ 12 meses post-IQ)

¿Ha acudido el paciente a la visita? ☐ Sí ☐ No

En caso **negativo**, ¿ha abandonado el paciente el estudio? ☐ Sí, cumplimentar hoja **Final del estudio**

☐ No, especificar razón.
- ☐1 Acontecimientos Adversos
- ☐2 Razones personales
- ☐3 Otros, especificar: ______________________

Fecha de visita 5: |__|__|__|__|__|__|__|__| (día mes año)

Cuestionario de Salud "Brief Pain Inventory" (Preguntas de 3 a 6)

INSTRUCCIONES:

Las preguntas que siguen se refieren a lo que usted piensa sobre su salud. Sus respuestas permitirán saber cómo se encuentra usted y hasta qué punto es capaz de hacer sus actividades habituales. Conteste cada pregunta tal y como se indica. Si no está seguro/a de cómo responder a una pregunta, por favor conteste lo que le parezca más cierto.

Fecha de realización: |__|__|__|__|__|__|__|__| (día mes año)

MARQUE UNA SOLA RESPUESTA

1. Clasifique su dolor haciendo un círculo alrededor del número que mejor describe la intensidad MÁXIMA de dolor sentido en las últimas 24 horas.

0	1	2	3	4	5	6	7	8	9	10
Ningún dolor										Máximo dolor

2. Clasifique su dolor haciendo un círculo alrededor del número que mejor describe la intensidad MÍNIMA de dolor sentido en las últimas 24 horas.

0	1	2	3	4	5	6	7	8	9	10
Ningún dolor										Máximo dolor

3. Clasifique su dolor haciendo un círculo alrededor del número que mejor describe la intensidad MEDIA de dolor sentido.

0	1	2	3	4	5	6	7	8	9	10
Ningún dolor										Máximo dolor

4. Clasifique su dolor haciendo un círculo alrededor del número que mejor describe la intensidad ACTUAL.

0	1	2	3	4	5	6	7	8	9	10
Ningún dolor										Máximo dolor

Cuestionario de Salud EQ-5D-3L

Marque con una cruz la respuesta de cada apartado que mejor describa su estado de salud el día de HOY.

Movilidad

No tengo problemas para caminar ❑

Tengo algunos problemas para caminar ❑

Tengo que estar en la cama ❑

Cuidado Personal

No tengo problemas con el cuidado personal ❑

Tengo algunos problemas para lavarme o vestirme ❑

Soy incapaz de lavarme o vestirme ❑

Actividades Cotidianas *(ej, trabajar, estudiar, hacer las tareas domésticas, actividades familiares o actividades durante el tiempo libre)*

No tengo problemas para realizar mis actividades cotidianas ❑

Tengo algunos problemas para realizar mis actividades cotidianas ❑

Soy incapaz de realizar mis actividades cotidianas ❑

Dolor / Malestar

No tengo dolor ni malestar ❑

Tengo moderado dolor o malestar ❑

Tengo mucho dolor o malestar ❑

Ansiedad / Depresión

No estoy ansioso ni deprimido ❑

Estoy moderadamente ansioso o deprimido ❑

Estoy muy ansioso o deprimido ❑

**MICROMANGUITO01**	|__|__| Nº Paciente	**Visita 6<br>(+ 12 meses post-IQ)**

Para ayudar a la gente a describir lo bueno o malo que es su estado de salud hemos dibujado una escala parecida a un termómetro en el cual se marca con un 100 el mejor estado de salud que pueda imaginarse y con un 0 el peor estado de salud que pueda imaginarse.

Nos gustaría que nos indicara en esta escala, en su opinión, lo bueno o malo que es su estado de salud en el día de HOY. Por favor, dibuje una línea desde el casillero donde dice "Su estado de salud hoy" hasta el punto del termómetro que en su opinión indique lo bueno o malo que es su estado de salud en el día de HOY.

Su estado de salud hoy

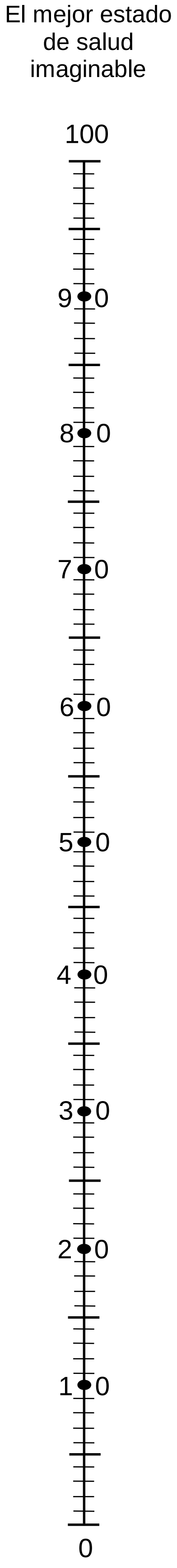

TEST DE CONSTANT-MURLEY:

A- DOLOR:

1- ¿Cuánto dolor tiene en el hombro en sus actividades de la vida diaria?	
0 puntos	Dolor severo o permanente
5 puntos	Dolor moderado
10 puntos	Dolor ligero
15 puntos	No dolor

2. Escala lineal:

Si "0" significa no tener dolor y "15" el mayor dolor que pueda sentir, haga un circulo sobre el nivel de dolor de su hombro.
La puntuación es inversamente proporcional a la la escala de dolor (Por ejemplo, un nivel de 5 son 10 puntos)

Nivel de dolor	0	1	2	3	4	5	6	7	8	9	10	11	12	13	14	15
Puntos	15	14	13	12	11	10	9	8	7	6	5	4	3	2	1	0

TOTAL A (1+2/2):

MICROMANGUITO01	Nº Paciente	Visita 6 (+ 12 meses post-IQ)

B- ACTIVIDADES DE LA VIDA DIARIA

1- ¿Está limitada la vida diaria por el hombro?

0 puntos	Limitación severa
2 puntos	Limitación moderada
4 puntos	Sin limitación

2- ¿Está limitada la vida deportiva por el hombro?

0 puntos	Limitación severa
2 puntos	Limitación moderada
4 puntos	Sin limitación

3- Sueño

0 puntos	El dolor impide dormir
1 punto	El dolor a veces despierta
2 puntos	Ninguna molestia

4- Altura a la que se puede elevar el brazo para coger un objeto

2 puntos	Altura de la cintura
4 puntos	Altura del xifoides
6 puntos	Altura del cuello
8 puntos	Altura de la cabeza
10 puntos	Encima de la cabeza

TOTAL B (1+2+3+4):

C- VALORACIÓN DEL PARÁMETRO MOVILIDAD

1- Flexión

0 puntos	0º - 30º
2 puntos	31º - 60º
4 puntos	61º - 90º
6 puntos	91º - 120º
8 puntos	121º - 150º
10 puntos	151º - 180º

2- Abducción

0 puntos	0º - 30º
2 puntos	31º - 60º
4 puntos	61º - 90º
6 puntos	91º - 120º
8 puntos	121º - 150º
10 puntos	151º - 180º

3- Rotación Externa

2 puntos	Mano detrás de la cabeza, codo adelante
4 puntos	Mano detrás de la cabeza, codo atrás
6 puntos	Mano sobre la cabeza, codo adelante
8 puntos	Mano sobre la cabeza, codo atrás
10 puntos	Elevación completa por encima de la cabeza

4- Rotación Interna

0 puntos	Dorso de la mano en trocanter
2 puntos	Dorso de la mano en la nalga
4 puntos	Dorso de la mano en articulación sacro-ilíaca
6 puntos	Dorso de la mano en la cintura
8 puntos	Dorso de la mano en la vértebra dorsal 12
10 puntos	Dorso de la mano en la zona interescapular

TOTAL C (1+2+3+4):

D- VALORACIÓN DEL PARÁMETRO FUERZA

La fuerza se medirá con un dinamómetro (mecánico o electrónico), con el muelle fijado al suelo por un extremo, y la cincha fijada a la muñeca del paciente por el otro, con la extremidad superior en abducción a 90º en el plano escapular, el codo extendido y el antebrazo pronado.
El resultado vendrá dado por la mayor de tres mediciones consecutivas de 5 segundos de duración. Si el paciente no alcanza los 90º, la fuerza se mide en la máxima abducción que pueda conseguir.
Se puntúa hasta un máximo de 25 puntos (12,5kg)
La medición debe de ser libre de dolor, si el paciente tiene dolor al hacer fuerza puntúa 0 puntos.

TOTAL D (Media Kg. x 2):

TOTAL (A + B + C + D):

E- VALORACIÓN DE LA RESONANCIA MAGNÉTICA

TENDON / ESTADO	NORMAL	TENDINOPATIA	ROTURA PARCIAL	ROTURA COMPLETA
SUPRAESPINOSO				
INFRAESPINOSO				
SUBESCAPULAR				

El radiólogo sobre el dibujo indica el estado de reparación de la huella y zona cubierta de esta con tendón curado

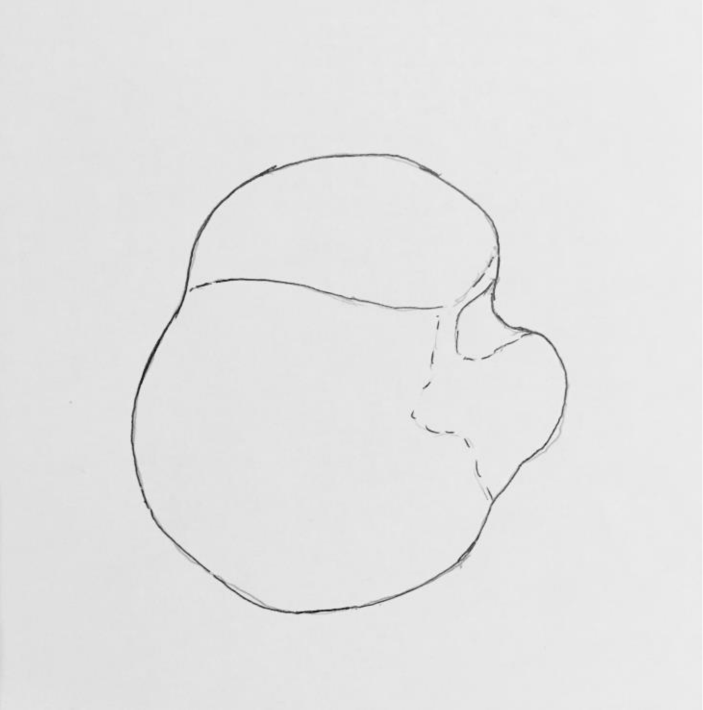

Medicación concomitante

¿El paciente ha recibido algún tratamiento desde la última visita? ☐ Sí ☐ No

En caso afirmativo, cumplimente la hoja de **medicación concomitante**.

Acontecimientos Adversos

¿El paciente ha presentado algún Acontecimiento Adverso desde la última visita? ☐ Sí ☐ No

En caso afirmativo, cumplimente la hoja de **Acontecimientos Adversos**.

Evaluación de complicaciones

¿El paciente ha presentado alguna complicación desde la última visita? ☐ Sí ☐ No

En caso afirmativo, definir el tipo de complicación y su tratamiento.

MICROMANGUITO01	Nº Paciente	Final del estudio

Final del estudio

¿Completó el paciente el estudio?: [ ] 0 = No 1 = Sí

Fecha de finalización o abandono: __ __ __ (día mes año)

Si **no**, especifique la razón principal de finalización precoz: [ ]

1 = Acontecimiento adverso(s) *(especificar en la hoja de AAs)*
2 = Ineficacia terapéutica
3 = Violación de protocolo
4 = Retirada del consentimiento por parte del paciente
5 = Pérdida de seguimiento
6 = Problemas administrativos
7 = Muerte *(por favor, complete el formulario de Acontecimientos Adversos Graves (A.A.G.) / Serious Adverse Events (SAE)).*

Fecha de la muerte: __ __ __ (día mes año)

Causa principal de la muerte: ______________________

(utilizar terminología médica precisa)

Por la presente declaro que la información del paciente perteneciente al estudio cuyo código de identificación figura en estas páginas es fiel reflejo de la realidad.

______________________ Firma investigador

__ __ __ Fecha Firma

**MICROMANGUITO01**	|___|___| Nº paciente	**Medicación Concomitante**

Medicación Concomitante

¿Ha tomado el paciente alguna medicación Concomitante durante el transcurso del estudio? ☐ No ☐ Sí, especificar

	**Medicación concomitante** *(usar nombre comercial si es posible)*	**Razón**	**Fecha de inicio** día / mes / año	*Marque si continúa en la eval. final*	***O:*** **Fecha final** día / mes / año
1			|__|__|__|	❑	|__|__|__|
2			|__|__|__|	❑	|__|__|__|
3			|__|__|__|	❑	|__|__|__|
4			|__|__|__|	❑	|__|__|__|
5			|__|__|__|	❑	|__|__|__|
6			|__|__|__|	❑	|__|__|__|
7			|__|__|__|	❑	|__|__|__|
8			|__|__|__|	❑	|__|__|__|
9			|__|__|__|	❑	|__|__|__|
10			|__|__|__|	❑	|__|__|__|

Nota: Por favor asegúrese que los datos de este formulario son consistentes con los datos de la Historia médica / Enfermedades concomitantes / Acontecimientos adversos.	*Marque aquí si esta es la última página de Medicación Concomitante* ☐

**MICROMANGUITO01**	Nº paciente	**Medicación Concomitante**

Medicación Concomitante (Continuación)

	**Medicación concomitante** *(usar nombre comercial si es posible)*	**Razón**	**Fecha de inicio** día / mes / año	*Marque si continúa en la eval. final*	***O:*** **Fecha final** día / mes / año
11				❑	
12				❑	
13				❑	
14				❑	
15				❑	
16				❑	
17				❑	
18				❑	
19				❑	
20				❑	

Nota: Por favor asegúrese que los datos de este formulario son consistentes con los datos de la Historia médica / Enfermedades concomitantes / Acontecimientos adversos.	*Marque aquí si esta es la última página de Medicación Concomitante* ☐

MICROMANGUITO01	Nº paciente	Acontecimientos Adversos

Acontecimientos Adversos

¿Ha tenido el paciente algún Acontecimiento Adverso durante el transcurso del estudio? ☐ No ☐ Sí, especificar

Por favor describa por línea un único AA para:

- *Todos los nuevos Acontecimientos Adversos (AAs)*
- *Todos los AAs que han aumentado su severidad*
- *Todos los AAs con cambios en relación con el fármaco*
- *Todas las enfermedades concomitantes presentes al inicio del estudio que hayan empeorado*

Severidad
1=Leve
2=Moderado
3=Severo

Relación con la medicación en estudio
0=No sospechada
1=Sospechada

Acción tomada *(entre uno o más códigos)*
0=Ninguna
1=Administracíón de medicación concomitante
2=Administración de tratamiento no farmacológico
3=Hospitalización / prolongación de la hospitalización

Marque si acont. adverso cumple definición de "grave"	**Acontecimiento Adverso**	**Severidad**	**Relación con la medicación en estudio**	**Fecha de inicio** día	mes	año	*Marque si continúa en la eval. final*	***O:*** **Fecha final** día	mes	año	**Acción tomada**
1 ❑							❑				
2 ❑							❑				
3 ❑							❑				
4 ❑							❑				
5 ❑							❑				
6 ❑							❑				
7 ❑							❑				

Cualquier Acontecimiento Adverso que cumple la definición de "grave" requiere notificación a TFS según está descrito en el protocolo y la cumplimentación de la hoja de "Acontecimiento Adverso Grave" (AAG).	*Si se ha tomado Medicación Concomitante, por favor complete la hoja de "Medicación Concomitantes".*	*Marque aquí si esta página es la última de Acontecimientos Adversos* ☐

MICROMANGUITO01	Nº paciente	Acontecimientos Adversos

Acontecimientos Adversos (Continuación)

Por favor describa por línea un único AA para:

- *Todos los nuevos Acontecimientos Adversos (AAs)*
- *Todos los AAs que han aumentado su severidad*
- *Todos los AAs con cambios en relación con el fármaco*
- *Todas las enfermedades concomitantes presentes al inicio del estudio que hayan empeorado*

Severidad
1=Leve
2=Moderado
3=Severo

Relación con la medicación en estudio
0=No sospechada
1=Sospechada

Acción tomada *(entre uno o más códigos)*
0=Ninguna
1=Administracíón de medicación concomitante
2=Administración de tratamiento no farmacológico
3=Hospitalización / prolongación de la hospitalización

	Marque si acont. adverso cumple definición de "grave"	**Acontecimiento Adverso**	**Severidad**	**Relación con la medicación en estudio**	**Fecha de inicio** día	mes	año	*Marque si continúa en la eval. final*	***O:*** **Fecha final** día	mes	año	**Acción tomada**
8	❑							❑				
9	❑							❑				
10	❑							❑				
11	❑							❑				
12	❑							❑				
13	❑							❑				
14	❑							❑				

Cualquier Acontecimiento Adverso que cumple la definición de "grave" requiere notificación a TFS según está descrito en el protocolo y la cumplimentación de la hoja de "Acontecimiento Adverso Grave" (AAG).	*Si se ha tomado Medicación Concomitante, por favor complete la hoja de "Medicación Concomitantes".*	*Marque aquí si esta página es la última de Acontecimientos Adversos* ☐

MICROMANGUITO01	Nº paciente	Acontecimientos Adversos

Acontecimientos Adversos (Continuación)

Por favor describa por línea un único AA para:

- *Todos los nuevos Acontecimientos Adversos (AAs)*
- *Todos los AAs que han aumentado su severidad*
- *Todos los AAs con cambios en relación con el fármaco*
- *Todas las enfermedades concomitantes presentes al inicio del estudio que hayan empeorado*

Severidad
1=Leve
2=Moderado
3=Severo

Relación con la medicación en estudio
0=No sospechada
1=Sospechada

Acción tomada *(entre uno o más códigos)*
0=Ninguna
1=Administracíón de medicación concomitante
2=Administración de tratamiento no farmacológico
3=Hospitalización / prolongación de la hospitalización

	Marque si acont. adverso cumple definición de "grave"	**Acontecimiento Adverso**	**Severidad**	**Relación con la medicación en estudio**	**Fecha de inicio** día	mes	año	*Marque si continúa en la eval. final*	***O:*** **Fecha final** día	mes	año	**Acción tomada**
15	❑							❑				
16	❑							❑				
17	❑							❑				
18	❑							❑				
19	❑							❑				
20	❑							❑				
21	❑							❑				

Cualquier Acontecimiento Adverso que cumple la definición de "grave" requiere notificación a TFS según está descrito en el protocolo y la cumplimentación de la hoja de "Acontecimiento Adverso Grave" (AAG).	*Si se ha tomado Medicación Concomitante, por favor complete la hoja de "Medicación Concomitantes".*	*Marque aquí si esta página es la última de Acontecimientos Adversos* ☐

MICROMANGUITO01	Nº paciente	Acontecimientos Adversos

Acontecimientos Adversos (Continuación)

Por favor describa por línea un único AA para:

- *Todos los nuevos Acontecimientos Adversos (AAs)*
- *Todos los AAs que han aumentado su severidad*
- *Todos los AAs con cambios en relación con el fármaco*
- *Todas las enfermedades concomitantes presentes al inicio del estudio que hayan empeorado*

Severidad
1=Leve
2=Moderado
3=Severo

Relación con la medicación en estudio
0=No sospechada
1=Sospechada

Acción tomada *(entre uno o más códigos)*
0=Ninguna
1=Administracíón de medicación concomitante
2=Administración de tratamiento no farmacológico
3=Hospitalización / prolongación de la hospitalización

	Marque si acont. adverso cumple definición de "grave"	**Acontecimiento Adverso**	**Severidad**	**Relación con la medicación en estudio**	**Fecha de inicio** día	mes	año	*Marque si continúa en la eval. final*	***O:*** **Fecha final** día	mes	año	**Acción tomada**
22	❑							❑				
23	❑							❑				
24	❑							❑				
25	❑							❑				
26	❑							❑				
27	❑							❑				
28	❑							❑				

Cualquier Acontecimiento Adverso que cumple la definición de "grave" requiere notificación a TFS según está descrito en el protocolo y la cumplimentación de la hoja de "Acontecimiento Adverso Grave" (AAG).	*Si se ha tomado Medicación Concomitante, por favor complete la hoja de "Medicación Concomitantes".*	*Marque aquí si esta página es la última de Acontecimientos Adversos* ☐
